# Multivariate colorimetric phenotyping reveals genetic loci associated with soybean seed coat pigmentation and epicatechin accumulation

**DOI:** 10.1007/s11032-026-01655-8

**Published:** 2026-03-26

**Authors:** Eunsoo Lee, Sewon Park, Yeon Ju An, Jungmin Ha

**Affiliations:** 1 Upland Crop Breeding Division, Rural Development Administration, National Institute of Crop and Food Science, Miryang, 50424 Republic of Korea; 2 Department of Agriculture, Agricultural Genomics, Seoul National University, Seoul, 08826 Republic of Korea; 3 Department of Agriculture, Forestry and Bioresources and Research Institute of Agriculture and Life Sciences, Seoul National University, Seoul, Republic of Korea; 4 Crop Genomics Laboratory, Plant Genomics and Breeding Institute, Seoul National University, Rm. 4105 Bldg. 200 CALS, 1 Gwanak-ro, Gwanak-gu, Seoul, 08826 Republic of Korea

**Keywords:** Soybean, (-)-epicatechin, Multivariate colorimetric traits, PCA, QTL mapping

## Abstract

Seed coat pigmentation in soybean is controlled by complex genetic mechanisms involving structural and regulatory genes in the flavonoid biosynthetic pathway. Although brown seed coats are often associated with epicatechin (EC) accumulation, visual classification alone cannot reliably predict EC content. To quantitatively characterize seed coat coloration and its relationship with EC accumulation, we evaluated multivariate colorimetric traits (L*, a*, and b* values in the CIELAB color space) in 235 recombinant inbred lines (RILs) derived from Jinpung (yellow seed coat) and IT109098 (greenish-brown seed coat). Principal component analysis (PCA) of L*, a*, and b* values revealed that genotypes with detectable EC were confined to specific regions of the multivariate color space, indicating that EC accumulation is associated with coordinated color balance rather than overall pigmentation intensity. RILs with high EC content showed significantly lower L* (32.76 ± 2.49) and b* (13.18 ± 2.66) values and higher a* values (5.47 ± 1.31) than those with low EC content. Quantitative trait loci (QTL) mapping identified thirteen loci associated with L*, a*, b*, and principal component scores across chromosomes 01, 05, 06, 08, and 19 A major locus on chromosome 08 near the classical *I* locus explained a large proportion of phenotypic variance in pigmentation traits. In addition, loci on chromosomes 06 and 19 were associated with integrated color components, suggesting quantitative modulation of EC accumulation. Candidate genes within these regions included flavonoid 3′-hydroxylase and transcription factors such as *MYB117*, *MYB60*, and *TCP5*, supported by sequence variation and differential expression analyses. These findings demonstrate that multivariate colorimetric traits provide a useful phenotyping framework for dissecting seed coat pigmentation and EC accumulation and for pre-selecting high-EC soybean lines.

**Supplementary Information:**

The online version contains supplementary material available at 10.1007/s11032-026-01655-8.

## Introduction

Soybean (*Glycine max* (L.) Merr.) exhibits diverse seed coat colors, including yellow, green, brown, black, and bicolor types, reflecting both domestication history and underlying genetic variation (Yuan et al. 2022). Seed coat pigmentation is primarily determined by the flavonoid biosynthetic pathway and is genetically controlled by multiple structural and regulatory loci. The classical *I* locus on chromosome 8, consisting of inverted repeat clusters of *chalcone synthase* (*CHS*) genes, plays a central role in the presence or absence of pigmentation through RNA silencing mechanisms (Cho et al. 2000, 2019). In addition to the *I* locus, other structural genes contribute to pigmentation diversity, including *flavonoid 3′-hydroxylase* (*F3′H*, *T* locus), *flavonoid 3′*,*5′-hydroxylase* (*F3′5′H*, *W1* locus), *flavanone 3-hydroxylase* (*F3H*, *Wp* locus), and *anthocyanidin reductase* (*ANR*, *O* locus) (Kovinich et al. 2011, 2012; Senda 2017; Zabala and Vodkin 2014). Among these, *CHS*, *F3′H*, and *ANR* are directly involved in the biosynthesis of epicatechin (EC), a flavan-3-ol monomer contributing to brown pigmentation in soybean seed coats (Kovinich et al. 2011; Song et al. 2016). EC accumulation in pigmented seed coats has been previously documented (Ha et al. 2018). Beyond structural genes, transcriptional regulation further modulates flavonoid accumulation. *MYB*, *bHLH*, and *WD40* transcription factors form ternary complexes that coordinately regulate genes in the flavonoid pathway, resulting in multilayered regulatory networks underlying seed coat coloration (Dixon et al. 2013; Lu et al. 2021).

As a terminal product of the flavonoid pathway, EC also serves as a monomeric unit of proanthocyanidins (Ha et al. 2018; Rauf et al. 2019). Beyond its role as a pigment, EC has attracted attention for its antioxidant, anti-inflammatory, and cardioprotective properties, underscoring its nutritional significance in soybean-derived foods (Gutierrez-Salmean et al. 2014; Bonetti et al. 2017; Prakash et al. 2019; Zbinden-Foncea et al. 2022). However, EC levels vary substantially among pigmented soybean accessions despite similar visual coloration, indicating that seed coat color alone does not reliably predict EC concentration (Ha et al. 2018; Jun et al. 2018; Lim et al. 2021; Lu et al. 2021). Accurate EC quantification relies on destructive biochemical assays such as high-performance liquid chromatography (HPLC) or chemical staining methods (Li et al. 1996; Ha et al. 2018). Although robust, these approaches are labor-intensive and less suitable for large-scale phenotyping. Improving phenotypic resolution using quantitative, non-destructive approaches may therefore facilitate more efficient genetic dissection of EC-associated traits.

Quantitative color measurement using the Commission Internationale de l’Eclairage (CIE) L*, a*, b* color space provides an objective framework for describing pigmentation, where L* indicates lightness, a* the red–green axis, and b* the yellow–blue axis (Ly et al. 2020). The CIELAB system has been applied in several crop species to quantify pigment-related variation and support genetic analyses of visually complex traits (Black and Panozzo 2004; Hossain et al. 2011; Sooriyapathirana et al. 2010). Unlike categorical scoring, L*, a*, and b* values represent correlated components within a three-dimensional color space. Because L, a, and b* values are continuous and partially correlated traits, they may capture subtle genetic effects that are masked in categorical pigmentation scores or single-metabolite measurements. Pigmentation intensity and hue are influenced by multiple biochemical factors, such that single-parameter measurements may not fully capture underlying metabolic variation. Multivariate approaches such as principal component analysis (PCA) enable dimensionality reduction while preserving major phenotypic axes. Integrating multidimensional colorimetric traits with genetic mapping may therefore provide complementary insights into how continuous color variation relates to EC accumulation and refine quantitative trait loci (QTL)-based dissection of pigmentation-associated traits.

Previous studies have identified loci associated with seed coat pigmentation and flavonoid-related traits in soybean through linkage mapping and association analyses (Song et al. 2016; Yuan et al. 2022). Major loci such as the *I* locus exert strong effects on pigmentation patterns, while additional structural genes contribute to flavonoid variation (Cho et al. 2019; Kovinich et al. 2011). Using the same recombinant inbred line (RIL) population derived from Jinpung × IT109098, two major QTLs for EC content, *qEC06* and *qEC08*, were previously identified through high-density linkage mapping (Park et al. 2024). That study demonstrated that quantitative EC accumulation could be genetically dissected independently of the classical pigmentation switch at the *I* locus. However, the phenotypic framework relied primarily on biochemical quantification of EC or categorical assessment of pigmentation. Re-examining the same genetic population using a quantitative, multivariate, and non-destructive colorimetric approach may offer complementary insights beyond conventional metabolite quantification, while preserving sample integrity. It remains unclear whether multivariate colorimetric traits can serve as genetically informative proxies for EC accumulation and thereby refine the interpretation of EC-associated QTLs.

In this study, we applied a quantitative, non-destructive, multivariate colorimetric phenotyping framework to the Jinpung × IT109098 RIL population to investigate the relationship between seed coat coloration and EC accumulation. L*, a*, and b* values were measured within the CIELAB color space, and PCA was applied to capture major axes of variation. QTL mapping was conducted to identify genomic regions associated with multivariate colorimetric traits and to examine their correspondence with previously identified EC loci. Sequence variation and gene expression analyses were further performed to identify candidate genes underlying the detected QTL regions. By integrating multidimensional phenotyping with genetic mapping, this study provides a complementary framework for interpreting the genetic architecture of seed coat pigmentation and flavonoid accumulation in soybean.

## Materials and methods

### Plant materials and epicatechin quantification

The RIL population consisting of 235 lines derived from a cross between Jinpung (yellow seed coat) and IT109098 (greenish-brown seed coat) was developed using the single-seed descent method as previously described (Park et al. 2024). Seeds were harvested from three plants per genotype over two consecutive growing seasons (2020 and 2021). EC content was previously quantified using high-performance liquid chromatography (HPLC) as reported in Park et al. (2024). Briefly, seed coats were separated, ground, and subjected to HPLC analysis to determine EC concentration. BLUP values of EC content across environments were used for correlation and comparative analyses in the present study.

### Determination of seed coat color and colorimetric measurements

Seed coat color of each genotype was classified into four categories (yellow, green, light brown, and brown) based on visual observation of 100 seeds per genotype in three biological replicates across two years. Objective color measurements were obtained using a CR-400 colorimeter (Konica Minolta Inc., Tokyo, Japan) in the CIELAB color space. L* (lightness), a* (red–green axis), and b* (yellow–blue axis) values were recorded for three biological replicates per genotype. Best linear unbiased prediction (BLUP) values were calculated using the lmer function in R v4.2.1 software to account for genotype, year, and genotype-by-environment interaction effects, with these factors treated as random.

### Principal component analysis and classification of EC-enriched regions

PCA was performed on BLUP values of L*, a*, and b* to reduce dimensionality and define integrated color variation. The number of principal components was determined based on the scree plot. Because EC content was not linearly associated with PC1 or PC2 values, PC ranges were empirically divided to identify regions enriched for EC-containing genotypes. PC1 values were grouped into five intervals and PC2 values into five intervals based on distribution patterns. RILs were subsequently classified into “low-EC” and “high-EC” groups according to PC ranges that showed significant differences in EC content. Statistical significance was evaluated using t-tests in R.

### Linkage map and QTL analysis

The high-density linkage map constructed from genotyping-by-sequencing data of Jinpung and IT109098 (Park et al. 2024) was used for QTL mapping in this study. Inclusive composite interval mapping implemented in QTL IciMapping v4.2 was applied using BLUP values of L*, a*, b*, PC1, and PC2. The scanning step was set to 0.5 cM. Significance thresholds were determined using 1,000 permutation tests at a type I error rate of 0.05.

### Identification of candidate genes within QTL regions

Sequence variation within QTL intervals was analyzed to identify candidate genes potentially responsible for the detected QTL effects. Protein-coding genes located within each QTL interval, including 2 kb upstream and downstream flanking regions, were extracted based on the soybean reference genome (Wm82.a2.v1). Resequencing data of the parental lines Jinpung and IT109098, previously generated (Park et al. 2024), were processed using Trimmomatic v0.39 (Bolger et al. 2014) for quality trimming. Clean reads were aligned to the Wm82.a2.v1 reference genome using Burrows-Wheeler Aligner (BWA-MEM) (Li 2013). Sequence variants were called using BCFtools mpileup. Variants with a quality score greater than 30 and read depth greater than 3 were retained using VCFtools. The functional impact of identified SNPs and InDels within QTL intervals was predicted using SnpEff (Cingolani et al. 2012). Putative gene functions were inferred based on *Arabidopsis thaliana* ortholog annotations obtained from TAIR (Araport11).

### RNA-seq data reanalysis and differential expression analysis

RNA-seq datasets from developing soybean seeds were reanalyzed to investigate gene expression patterns within QTL regions. Sequence Read Archive (SRA) files corresponding to Hwangkeum (yellow seed coat), IT109098 (greenish-brown seed coat), and IT182932 (black seed coat) at R5 and R7 developmental stages were downloaded from NCBI SRA as originally reported by Ha et al. (2018). Accession numbers were SRR5838836 and SRR5838835 for Hwangkeum, SRR5838834 and SRR5838833 for IT109098, and SRR5838832 and SRR5838831 for IT182932. RNA files were converted to FASTQ format using the sra-toolkit (Leinonen et al. 2011). Low-quality reads and adapter sequences were removed using Trimmomatic v0.39 (Bolger et al. 2014). Cleaned reads were aligned to the soybean reference genome (Wm82.a2.v1) using HISAT2 (Kim et al. 2019). Gene-level read counts were obtained using FeatureCounts (Liao et al. 2014). Raw counts were filtered to retain genes with counts greater than 10 and normalized using the trimmed mean of M-values (TMM) method implemented in edgeR (Robinson et al. 2010). Differentially expressed genes (DEGs) were defined as those showing |log2 fold change| ≥ 1 between Hwangkeum and IT109098 or between Hwangkeum and IT182932 at either R5 or R7 stages.

## Results

### Phenotypic architecture of multivariate colorimetric traits

**Fig. 1:**
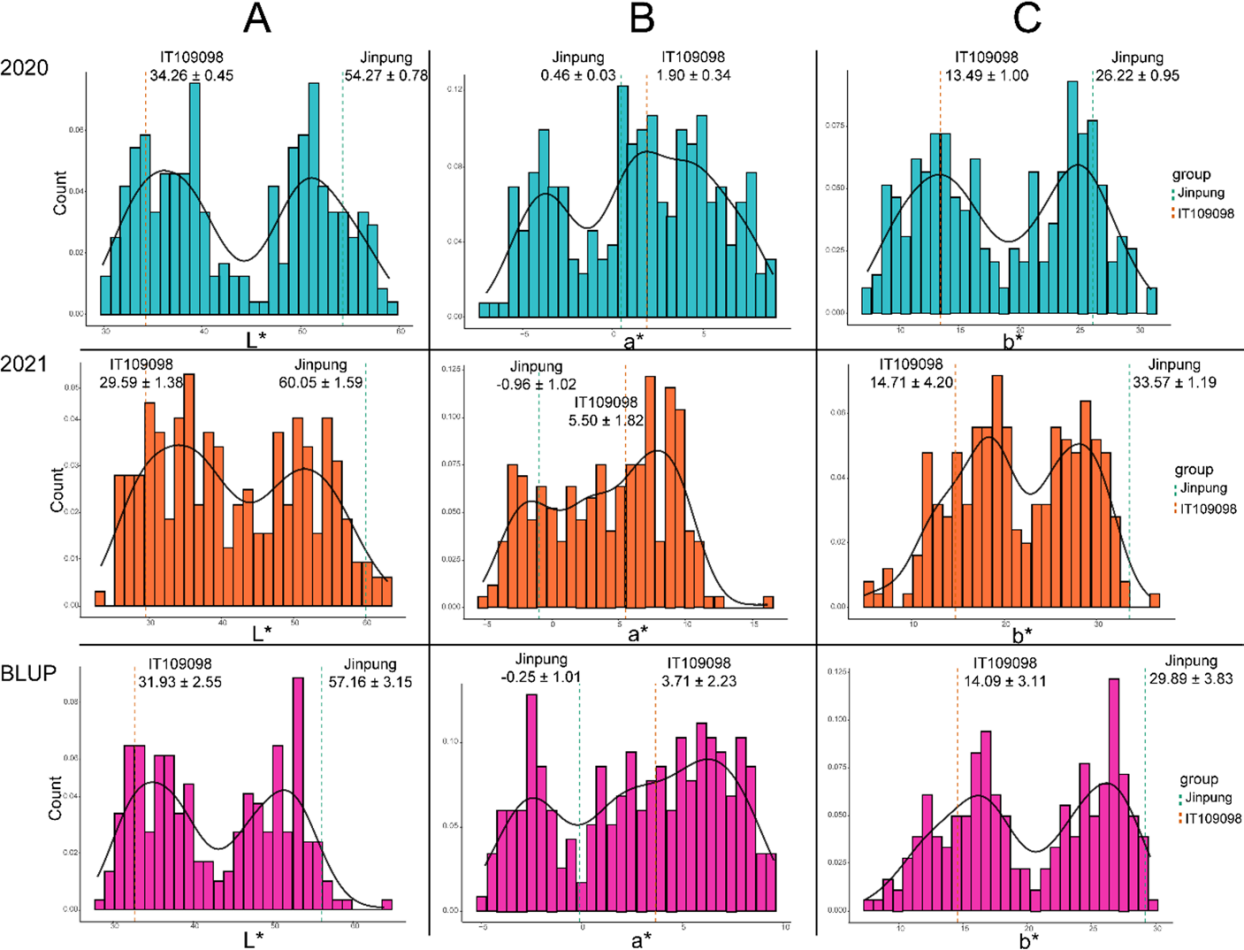
Distribution of seed coat colors of recombinant inbred lines (RILs) derived from Jinpung x IT109098 in Commission Internationale de l’Eclairage (CIE) L*, a*, b* color space. (**A**) L* values for 2020, 2021, and the best linear unbiased prediction (BLUP). (**B**) a* values for 2020, 2021, and BLUP. (**C**) b* values for 2020, 2021 and BLUP; dashed lines indicated parental phenotypes

The RIL population derived from Jinpung × IT109098 exhibited diverse seed coat colors, including yellow, green, light brown, and brown (Fig. S1). To quantitatively characterize this variation, seed coat coloration was measured using L*, a*, and b* values within the CIELAB color space. The distribution of BLUP values demonstrated substantial phenotypic variation among the 235 RILs (Table S1; Fig. 1). BLUP values ranged from 28.09 to 63.89 for L*, − 4.79 to 9.53 for a*, and 7.42 to 29.59 for b*, indicating broad quantitative segregation of color traits within the population. Broad-sense heritability (*H²*) estimates were high for L* (0.90), a* (0.88), and b* (0.80), confirming that colorimetric traits are predominantly genetically controlled and suitable for genetic analysis. EC content in this RIL population, previously quantified using HPLC (Park et al. 2024), ranged from 0.00 to 1965.33 µg/g and exhibited high heritability across environments. This substantial quantitative variation provided the basis for examining the relationship between colorimetric traits and EC accumulation. Correlation analysis revealed strong interrelationships among the three color parameters (Fig. S2). L* and b* values were highly positively correlated (*r* = 0.95), while both showed significant negative correlations with a* (*r* = − 0.70 and − 0.74, respectively). EC content showed moderate but significant correlations with colorimetric traits, with negative correlations for L* (*r* = − 0.47) and b* (*r* = − 0.46), and a positive correlation for a* (*r* = 0.22) (*P* < 0.001). These results indicate that darker and less yellow seed coats tend to be associated with higher EC content, but the moderate magnitude of these correlations suggests that individual color parameters alone do not fully explain EC variation. Together, these findings demonstrate that seed coat coloration in the RIL population is genetically stable, quantitatively distributed, and structured across correlated color axes, providing a foundation for multivariate analysis of pigmentation-associated traits.

### Multivariate color space reveals EC-enriched regions

**Fig. 2:**
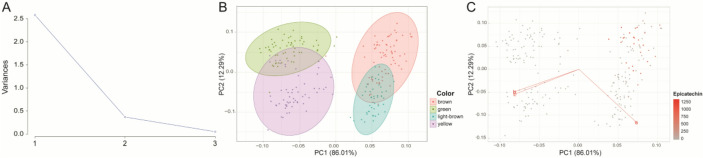
Principal component analysis (PCA) on L*, a* and b* values. (**A**) Scree plot for determining the number of PCs. (**B**) PCA on L*, a*, b* values and clustering of seed coat color in 4 groups (yellow, green, light brown, and brown) based on the actual observation. (**C**) PCA on L*, a*, b* values with their axes on the PC coordinates and identification of recombinant inbred lines (RILs) with epicatechin (EC) content

**Fig. 3:**
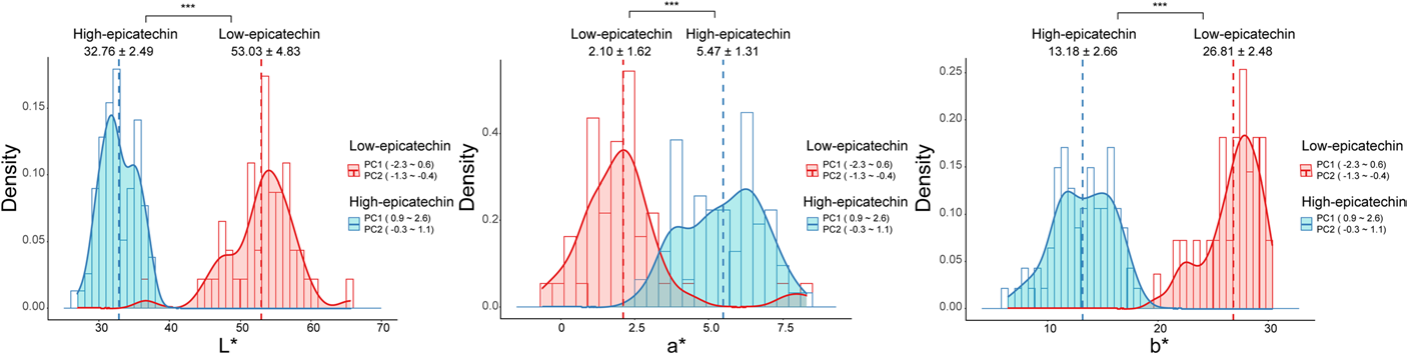
Distribution of L*, a*, and b* values upon significance of EC content between ranges of principal component (PC) values (PC1 and PC2). ***: P ≤ 0.001

PCA was performed to summarize correlated variation among L*, a*, and b* values within a reduced multivariate color space. The scree plot indicated that two components captured most of the phenotypic variation (Fig. 2A). PC1 and PC2 together explained 98.30% of the total variance, accounting for 86.01% and 12.29%, respectively (Fig. 2B), and were therefore used to represent the primary axes of seed coat color variation. Projection of the RILs onto the PC1–PC2 space showed clustering consistent with visually observed seed coat color categories (Fig. 2C). Overlaying EC content onto this space revealed that genotypes with detectable EC were primarily located in the upper-right region of the PC1–PC2 space, corresponding to positive PC1 values and near-zero to positive PC2 values. Although only brown-seeded lines accumulated EC, not all brown-seeded RILs contained detectable EC, indicating that categorical color classification alone does not sufficiently explain EC variation. PC1 and PC2 values were not linearly associated with EC content (Fig. S3), and regression analysis restricted to brown-seeded lines showed weak predictive relationships (Fig. S4), indicating that PC scores do not directly predict EC concentration. Despite this absence of linearity, genotypes with detectable EC were concentrated within defined PC1–PC2 regions. Based on this distribution, RILs were classified into low- and high-EC groups using specific PC ranges, with low-EC lines having PC1 values from − 2.6 to 0.6 and PC2 values from − 1.3 to − 0.4, and high-EC lines having PC1 values from 0.9 to 2.6 and PC2 values from − 0.3 to 1.1 (Fig. S5). These groups differed significantly in L*, a*, and b* values (*P* < 0.001; Table S2), demonstrating that multivariate colorimetric parameters effectively discriminate EC-enriched phenotypic regions (Fig. 3). Importantly, PC1 and PC2 represent integrated axes summarizing coordinated variation among L*, a*, and b*, rather than independent traits. Thus, EC variation is interpreted within a multidimensional phenotypic space instead of through isolated color parameters, providing a structured basis for subsequent QTL analysis.

### QTL mapping of integrated color components identifies major and additional loci

**Fig. 4:**
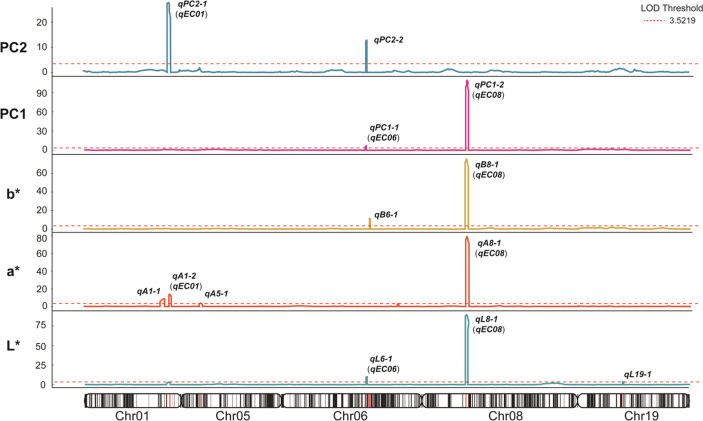
Positions of quantitative trait loci (QTLs) associated with seed coat color. Logarithm of odds (LOD) scores above the threshold value of 3.5219 indicate the position of QTL regions on the linkage map

QTL mapping was performed using BLUP values of L, a, b*, and principal component (PC) scores, based on a previously constructed high-density linkage map for the Jinpung × IT109098 RIL population (Park et al. 2024). In total, thirteen significant QTLs associated with integrated color components were detected across chromosomes 01, 05, 06, 08, and 19 (Table 1; Fig. 4). A major locus on chromosome 08 was consistently detected across L*, a*, b*, and PC1. This region showed exceptionally high LOD values, reaching 89.22 for L*, 78.76 for a*, 75.75 for b*, and 109.31 for PC1, and explained a substantial proportion of phenotypic variance (73.68% for L*, 70.62% for a*, 72.14% for b*, and 82.75% for PC1). These values clearly indicate that this locus exerts a dominant effect on seed coat pigmentation. The physical interval corresponds to the region near the classical *I* locus (~ 200 kb), confirming its central role in structuring overall color variation within the integrated phenotypic space. Beyond this major locus, several additional QTLs contributed to quantitative variation in color components. On chromosome 01, the *qEC01* region was associated with both a* and PC2 variation. In particular, the PC2-associated locus *qPC2-1* exhibited a LOD value of 27.86 and explained 35.94% of phenotypic variance, indicating a relatively strong secondary effect compared to other minor loci. The a*-associated locus within the same interval (LOD = 13.78; PVE = 6.01%) also suggests a role in regulating color balance. On chromosome 06, *qB6-1* was detected for b* with a LOD value of 11.62 and explained 5.21% of variance, indicating a moderate contribution to yellow–blue axis variation. In addition, *qPC2-2* on chromosome 06, associated with PC2, showed a LOD value of 12.83 and accounted for 13.84% of phenotypic variance, supporting its role in shaping coordinated variation along the second principal axis. The *qL6-1* region, overlapping the previously reported *qEC06* interval, showed a modest effect (LOD = 10.13; PVE = 3.77%), suggesting quantitative modulation of pigmentation intensity. On chromosome 19, *qL19-1* was associated with L* variation, although it explained a relatively small proportion of phenotypic variance (LOD = 3.80; PVE = 1.33%). Despite its modest effect size, this locus is of particular interest due to the presence of transcription factor candidates within the interval. Other detected loci on chromosomes 01 and 05 showed comparatively minor effects and explained limited proportions of variance. Collectively, these results indicate that seed coat coloration within the integrated phenotypic space is primarily governed by a dominant locus near the *I* region, while several additional loci, such as *qL19-1*, *qEC01*, *qB6-1*, and *qPC2-2*, contribute to quantitative modulation of pigmentation-related traits.

**Table 1:** Quantitative trait loci (QTLs) for seed coat color in L*, a*, b*, and principal component (PC) values identified by inclusive composite interval mapping of 235 RILs derived from Jinpung x IT109098

Trait	Locus	Chr.	Interval (cM)	Flanking markers	LOD	PVE ^a^ (%)	Add. ^b^	Source of beneficial allele	No. of genes
L*	*qL6-1* (*qEC06*)	6	161.75–163.75	Chr06_18555043, Chr06_19081766	10.13	3.77	1.76	Jinpung	22
	*qL8-1 * *(qEC08)*	8	85.75–86.75	Chr08_8771172, Chr08_9214678	89.22	73.68	7.79	Jinpung	50
	*qL19-1*	19	85.75–88.25	Chr19_37055654, Chr19_37703452	3.8	1.33	1.05	Jinpung	40
a*	*qA1-1*	1	149.25–155.75	Chr01_51796650, Chr01_52651673	8.57	3.6	0.73	Jinpung	98
	*qA1-2* *(qEC01)*	1	162.25–165.75	Chr01_53284324, Chr01_53808282	13.78	6.01	0.94	Jinpung	68
	*qA5-1*	5	37.25–41.25	Chr05_3769727, Chr05_4106987	3.94	1.52	0.48	Jinpung	39
	*qA8-1* *(qEC08)*	8	85.75–86.75	Chr08_8771172, Chr08_9214678	78.76	70.62	−3.22	IT109098	50
b*	*qB6-1*	6	169.25–170.75	Chr06_19968177, Chr06_21252730	11.62	5.21	1.36	Jinpung	50
	*qB8-1* *(qEC08)*	8	85.75–86.75	Chr08_8771172, Chr08_9214678	75.75	72.14	5.09	Jinpung	50
PC1	*qPC1-1* *(qEC06)*	6	161.75–163.75	Chr06_18555043, Chr06_19081766	6.86	2.00	−0.23	IT109098	22
	*qPC1-2* *(qEC08)*	8	85.75–86.75	Chr08_8771172, Chr08_9214678	109.31	82.75	−1.52	IT109098	50
PC2	*qPC2-1* *(qEC01)*	1	162.25–165.25	Chr01_53284324, Chr01_53808282	27.86	35.94	−0.37	IT109098	68
	*qPC2-2*	6	165.25–166.75	Chr06_19091784, Chr06_19310302	12.83	13.84	−0.23	IT109098	9

^a^ PVE: the percentage of phenotypic variance explained

^b^ Add: the allelic additive effect

### Candidate genes within major and additional QTL regions

To explore candidate genes underlying the detected QTLs, annotated genes within each QTL interval were surveyed and coding-sequence variation between Jinpung and IT109098 was examined (Tables S3 and S4). In addition, RNA-seq data from developing seeds at R5 and R7 stages of Hwangkeum (yellow seed coat), IT109098 (greenish-brown seed coat), and IT182932 (black seed coat), previously reported by Ha et al. (2018), were reanalyzed to identify differentially expressed genes (DEGs) within each QTL interval. The major chromosome 08 interval overlaps the classical *I* locus and showed the strongest statistical support among all detected QTLs. Given its dominant effect on pigmentation, further candidate exploration was focused on additional QTL regions contributing to quantitative variation beyond this primary locus. Within the *qL19-1* interval on chromosome 19, *Glyma.19G118500* and *Glyma.19G119300*, corresponding to *MYB117* and *MYB60*, respectively, were identified. Coding-sequence variation resulting in an amino acid substitution was detected in *Glyma.19G119300* between the parental lines (Table S4), and genotype-dependent differences in transcript abundance during R5 and R7 stages were observed (Fig. S6). The *qEC01* interval on chromosome 01 and the *qB6-1* region on chromosome 06, associated with PC2 and b* variation, respectively, both contained genes exhibiting sequence variation. Several genes within these intervals showed notable expression differences between yellow- and colored-seeded genotypes (Figs. S7 and S8). The *qPC2-2* interval contained *Glyma.06G204300* (*TCP5*), which showed sequence variation and expression differences among seed coat color genotypes (Fig. S9). Together, these results identify genes within *qL19-1*, *qEC01*, *qB6-1*, and *qPC2-2* intervals that exhibit sequence variation and/or differential expression during seed development.

## Discussion

### Integrated multivariate color space captures EC-associated variation

The present study demonstrates that seed coat pigmentation and EC accumulation are more effectively interpreted within an integrated multivariate framework than through isolated color parameters. Although L*, a*, and b* values individually showed significant correlations with EC content, the correlation coefficients were moderate, reaching − 0.47 for L*, − 0.46 for b*, and 0.22 for a*. These results indicate that no single color axis sufficiently explains EC variation. While EC accumulation was restricted to brown-seeded RILs, not all brown-seeded lines contained detectable EC, confirming that visual classification alone is insufficient for accurate inference. This distinction indicates that pigmentation intensity and metabolic accumulation are related but genetically separable traits. Previous studies have shown that EC contributes to brown pigmentation in soybean seed coats (Ha et al. 2018; Rauf et al. 2019; Tan et al. 2020), and the present results extend this understanding by revealing the complexity of its quantitative regulation. PCA clarified this relationship, with PC1 and PC2 explaining 86.01% and 12.29% of the total color variance, respectively, accounting for 98.30% cumulatively. Linear regression between PC scores and EC content yielded relatively weak associations, with *R* = 0.28 for PC1 and *R* = 0.47 for PC2, further indicating that EC accumulation is not linearly predicted by any single principal axis. Instead, genotypes with detectable EC were concentrated within a defined region of the PC1–PC2 space. This pattern indicates that EC enrichment is associated with coordinated shifts across multiple color components rather than variation along a single axis. Multivariate analytical approaches are widely used in metabolomics to interpret coordinated variation among correlated metabolites rather than relying on individual compound measurements (Fiehn 2002; Trygg and Wold 2002). In this context, the colorimetric multivariate framework applied here functions as a phenotypic analogue of metabolic fingerprinting, enabling non-destructive structured interpretation of complex pigmentation traits.

### A hierarchical genetic architecture of pigmentation

QTL mapping revealed a hierarchical genetic structure underlying seed coat coloration. The chromosome 08 locus located near the classical *I* region exhibited exceptionally high LOD values and explained more than 70% of phenotypic variance for individual color parameters and over 80% for PC1. The *I* locus is known to regulate pigmentation through RNA-mediated silencing of *CHS* genes (Cho et al. 2000, 2019), and structural genes such as *CHS*, *F3′H*, and *ANR* are central components of the EC biosynthetic pathway (Kovinich et al. 2011, 2012; Senda 2017; Zabala and Vodkin 2014). The magnitude of the chromosome 08 effect confirms its role as the primary pigmentation determinant within the integrated phenotypic space.

However, not all brown-seeded lines have high EC content, even though brown seed coats generally accumulate higher levels of EC. This indicates that pigmentation is not explained by a single genetic switch. Consistent with this complexity, additional loci contributed to quantitative modulation of pigmentation. The *qPC2-1* locus within the *qEC01* interval explained 35.94% of PC2 variance, and *qPC2-2* accounted for 13.84% of PC2 variation. The *qB6-1* locus explained 5.21% of variance for b*. Although smaller than the dominant chromosome 08 locus, these effects were statistically significant and collectively shaped coordinated variation within the multivariate color space. Together, these results support a multi-layered regulatory model in which a major pigmentation locus establishes baseline coloration, while multiple secondary loci fine-tune quantitative differences in color balance and associated flavonoid accumulation, reflecting a complex and integrated genetic control of seed coat pigmentation.

### Regulatory candidates associated with quantitative modulation

Candidate gene analysis identified transcription factor genes within key modulatory loci. Within the *qL19-1* interval, *Glyma.19G119300*, corresponding to *MYB60*, exhibited a missense variant between the parental lines and showed differential expression during seed development. *R2R3-MYB* transcription factors are established regulators of flavonoid biosynthesis and anthocyanin accumulation across plant species (Stracke et al. 2001; Dubos et al. 2010; Dixon et al. 2013; Lu et al. 2021). The presence of *MYB* genes within this interval, together with coding change and expression differences, supports their involvement in pigmentation-associated variation. The *qPC2-2* region contains *TCP5*. *TCP* transcription factors have been implicated in the regulation of anthocyanin-associated pathways through interactions with phytochrome signaling components (Han et al. 2019; Liu et al. 2021). Because PC2 captured variation in relative color balance rather than overall pigmentation intensity, the association of *TCP5* with PC2 variation suggests that shifts along the second principal axis may reflect coordinated regulatory influences on integrated color balance. Although *qEC01* did not contain a canonical structural enzyme directly linked to EC biosynthesis, multiple genes within this interval exhibited sequence variation and differential expression. Its relatively large contribution to PC2 variance indicates that regulation of color balance may involve regulatory or metabolic factors beyond core structural genes.

### Implications for non-destructive and image-based phenotyping

Compositional traits such as EC content require destructive biochemical assays including HPLC and DMACA-based methods (Ha et al. 2018; Li et al. 1996; Lu et al. 2021). In contrast, colorimetric measurements are non-destructive and rapid. By identifying EC-enriched regions within the PC1–PC2 space, this study demonstrates that integrated color indices can narrow the candidate pool prior to destructive validation, reducing labor and cost in breeding programs. Overall, loci identified through this colorimetric framework showed substantial overlap with those reported in previous transcriptomic and destructive QTL studies, underscoring the robustness of the approach. In addition, the multivariate phenotype space revealed regulatory variation affecting color balance that had been less apparent in earlier analyses. This framework aligns conceptually with advances in hyperspectral imaging and high-throughput phenotyping, where high-dimensional spectral or image-derived signals are integrated to infer complex physiological traits (Araus and Cairns 2014; Mahlein 2016). As digital agriculture expands, defining traits within structured multivariate spaces may enhance non-destructive prediction of metabolite-associated phenotypes in soybean and other crops.

## Supplementary Information

Below is the link to the electronic supplementary material.

Supplementary Material 1.

## Data Availability

All data generated or analysed during this study are included in this published article and its supplementary information file.

## Abbreviations

EC: Epicatechin
QTL: Quantitative trait locus
RIL: Recombinant inbred line
PCA: Principal component analysis
BLUP: The best linear unbiased prediction

## References

[CR1] Araus JL, Cairns JE (2014) Field high-throughput phenotyping: the new crop breeding frontier. Trends Plant Sci 19:52–61. 10.1016/j.tplants.2013.09.008 24139902 10.1016/j.tplants.2013.09.008

[CR2] Black CK, Panozzo JF (2004) Accurate technique for measuring color values of grain and grain products using a visible–NIR instrument. Cereal Chem 81:469–474. 10.1094/CCHEM.2004.81.4.469

[CR3] Bolger AM, Lohse M, Usadel B (2014) Trimmomatic: a flexible trimmer for Illumina sequence data. Bioinformatics 30:2114–2120. 10.1093/bioinformatics/btu170 24695404 10.1093/bioinformatics/btu170 PMC4103590

[CR4] Bonetti F, Brombo G, Zuliani G (2017) Nootropics, functional foods, and dietary patterns for prevention of cognitive decline. In: Watson RR (ed) Nutrition and functional foods for healthy aging. Academic Press, pp 211–232. 10.1016/B978-0-12-805376-8.00019-8

[CR5] Cho HJ, Farrand SK, Noel GR, Widholm JM (2000) High-efficiency induction of soybean hairy roots and propagation of the soybean cyst nematode. Planta 210:195–204. 10.1007/PL00008126 10664125 10.1007/PL00008126

[CR6] Cho YB, Jones SI, Vodkin LO (2019) Nonallelic homologous recombination events responsible for copy number variation within an RNA silencing locus. Plant Direct 3:e00162. 10.1002/pld3.162 31468028 10.1002/pld3.162 PMC6710647

[CR7] Cingolani P, Platts A, Wang LL et al (2012) A program for annotating and predicting the effects of single nucleotide polymorphisms. SnpEff Fly 6:80–92. 10.4161/fly.19695 22728672 10.4161/fly.19695 PMC3679285

[CR8] Dixon RA, Liu C, Jun JH (2013) Metabolic engineering of anthocyanins and condensed tannins in plants. Curr Opin Biotechnol 24:329–335. 10.1016/j.copbio.2012.07.004 22901316 10.1016/j.copbio.2012.07.004

[CR9] Dubos C, Stracke R, Grotewold E, Weisshaar B, Martin C, Lepiniec L (2010) MYB transcription factors in Arabidopsis. Trends Plant Sci 15:573–581. 10.1016/j.tplants.2010.06.005 20674465 10.1016/j.tplants.2010.06.005

[CR10] Fiehn O (2002) Metabolomics – the link between genotypes and phenotypes. Plant Mol Biol 48:155–171. 10.1023/A:1013713905833 11860207

[CR11] Gutierrez-Salmean G, Ciaraldi TP, Nogueira L et al (2014) Effects of (-)-epicatechin on molecular modulators of skeletal muscle growth and differentiation. J Nutr Biochem 25:91–94. 10.1016/j.jnutbio.2013.09.007 24314870 10.1016/j.jnutbio.2013.09.007 PMC3857584

[CR12] Ha J, Kim M, Kim MY et al (2018) Transcriptomic variation in proanthocyanidin biosynthesis pathway genes in soybean. J Sci Food Agric 98:2138–2146. 10.1002/jsfa.8698 28960323 10.1002/jsfa.8698

[CR13] Han X, Yu H, Yuan R et al (2019) Arabidopsis transcription factor TCP5 controls plant thermomorphogenesis by positively regulating PIF4 activity. iScience 15:611–622. 10.1016/j.isci.2019.04.005 31078552 10.1016/j.isci.2019.04.005 PMC6548983

[CR14] Hossain MA, Panozzo JF, Pittock C, Ford R (2011) Quantitative trait loci analysis of seed coat color components in chickpea. Can J Plant Sci 91:49–55. 10.4141/cjps10112

[CR15] Jun JH, Xiao X, Rao X, Dixon RA (2018) Proanthocyanidin subunit composition determined by functionally diverged dioxygenases. Nat Plants 4:1034–1043. 10.1038/s41477-018-0292-9 30478357 10.1038/s41477-018-0292-9

[CR16] Kim D, Paggi JM, Park C, Bennett C, Salzberg SL (2019) HISAT2 and HISAT-genotype. Nat Biotechnol 37:907–915. 10.1038/s41587-019-0201-4 31375807 10.1038/s41587-019-0201-4 PMC7605509

[CR17] Kovinich N, Saleem A, Arnason JT, Miki B (2011) Combined analysis of transcriptome and metabolite data reveals differences between black and brown soybean seed coats. BMC Genomics 12:381. 10.1186/1471-2164-12-381 21801362 10.1186/1471-2164-12-381 PMC3163566

[CR18] Kovinich N, Saleem A, Arnason JT, Miki B (2012) Identification of two anthocyanidin reductase genes and reduced proanthocyanidin accumulation. J Agric Food Chem 60:574–584. 10.1021/jf2033939 22107112 10.1021/jf2033939

[CR19] Leinonen R, Sugawara H, Shumway M (2011) The sequence read archive. Nucleic Acids Res 39:D19–D21. 10.1093/nar/gkq1019 21062823 10.1093/nar/gkq1019 PMC3013647

[CR41] Liao Y, Smyth GK, Shi W (2014) featureCounts: an efficient general purpose program for assigning sequence reads to genomic features. Bioinformatics 30(7):923–930. 10.1093/bioinformatics/btt656 10.1093/bioinformatics/btt656 24227677

[CR20] Li H (2013) Aligning sequence reads, clone sequences and assembly contigs with BWA-MEM. arXiv:1303.3997. https://arxiv.org/abs/1303.3997

[CR22] Lim YJ, Kwon SJ, Qu S, Kim DG, Eom SH (2021) Antioxidant contributors in seed, seed coat, and cotyledon of γ-ray-induced soybean mutant lines with different seed coat colors. Antioxidants 10:353. 10.3390/antiox10030353 33652948 10.3390/antiox10030353 PMC7996878

[CR23] Liu Z, Wang Y, Fan K, Li Z, Jia Q, Lin W, Zhang Y (2021) PHYTOCHROME-INTERACTING FACTOR 4 (PIF4) negatively regulates anthocyanin accumulation by inhibiting PAP1 transcription in Arabidopsis seedlings. Plant Sci 303:110788. 10.1016/j.plantsci.2020.110788 33487363 10.1016/j.plantsci.2020.110788

[CR21] Li YG, Tanner G, Larkin P (1996) The DMACA–HCl protocol and the threshold proanthocyanidin content for bloat safety in forage legumes. J Sci Food Agric 70(19960170:189):89–101. 10.1002/(SICI)1097-0010. ::AID-JSFA470>3.0.CO;2-N

[CR24] Lu N, Rao X, Li Y, Jun JH, Dixon RA (2021) Dissecting the transcriptional regulation of proanthocyanidin and anthocyanin biosynthesis in soybean (Glycine max). Plant Biotechnol J 19:1429–1442. 10.1111/pbi.13562 33539645 10.1111/pbi.13562 PMC8313137

[CR25] Ly BCK, Dyer EB, Feig JL, Chien AL, Del Bino S (2020) Research techniques made simple: cutaneous colorimetry: a reliable technique for objective skin color measurement. J Invest Dermatology 140(1):3–12e1. 10.1016/j.jid.2019.11.003 10.1016/j.jid.2019.11.003 31864431

[CR26] Mahlein AK (2016) Plant disease detection by imaging sensors – parallels and specific demands for precision agriculture and plant phenotyping. Plant Dis 100:241–251. 10.1094/PDIS-03-15-0340-FE 30694129 10.1094/PDIS-03-15-0340-FE

[CR27] Park S, Kwon H, Park GT et al (2024) Selective allele stacking of a novel quantitative trait locus facilitates enhancement of seed epicatechin content in soybean. Euphytica 220:90. 10.1007/s10681-024-03345-y

[CR28] Prakash M, Basavaraj BV, Murthy KC (2019) Biological functions of epicatechin: plant cell to human cell health. J Funct Foods 52:14–24. 10.1016/j.jff.2018.10.021

[CR29] Rauf A, Imran M, Abu-Izneid T, Patel S, Pan X, Naz S, Rasul SH (2019) Proanthocyanidins: a comprehensive review. Biomed Pharmacother 116:108999. 10.1016/j.biopha.2019.108999 31146109 10.1016/j.biopha.2019.108999

[CR30] Robinson MD, McCarthy DJ, Smyth GK (2010) edgeR: a Bioconductor package for differential expression analysis of digital gene expression data. Bioinformatics 26:139–140. 10.1093/bioinformatics/btp616 19910308 10.1093/bioinformatics/btp616 PMC2796818

[CR31] Senda M, Yamaguchi N, Hiraoka M et al (2017) Accumulation of proanthocyanidins and/or lignin deposition in buff-pigmented soybean seed coats may lead to frequent defective cracking. Planta 245:659–670. 10.1007/s00425-016-2624-5 27995313 10.1007/s00425-016-2638-8

[CR32] Song J, Liu Z, Hong H, Ma Y, Tian L, Li X, Qiu LJ (2016) Identification and validation of loci governing seed coat color by combining association mapping and bulk segregation analysis in soybean. PLoS ONE 11:e0159064. 10.1371/journal.pone.0159064 27404272 10.1371/journal.pone.0159064 PMC4942065

[CR33] Sooriyapathirana SS, Khan A, Sebolt AM, Wang D, Bushakra JM, Lin-Wang K, Allan AC, Gardiner SE, Chagné D (2010) QTL analysis and candidate gene mapping for skin and flesh color in sweet cherry fruit (Prunus avium L). Tree Genet Genomes 6:821–832. 10.1007/s11295-010-0293-1

[CR34] Stracke R, Werber M, Weisshaar B (2001) The R2R3-MYB gene family in Arabidopsis thaliana. Curr Opin Plant Biol 4:447–456. 10.1016/S1369-5266(00)00199-0 11597504 10.1016/s1369-5266(00)00199-0

[CR35] Tan J, de Bruijn WJ, van Zadelhoff A, Lin Z, Vincken JP (2020) Browning of epicatechin (EC) and epigallocatechin (EGC) by auto-oxidation. J Agric Food Chem 68:13879–13887. 10.1021/acs.jafc.0c05063 33171045 10.1021/acs.jafc.0c05716 PMC7705966

[CR36] Trygg J, Wold S (2002) Orthogonal projections to latent structures (O-PLS). J Chemometrics 16:119–128. 10.1002/cem.695

[CR37] Yuan B, Yuan C, Wang Y et al (2022) Identification of genetic loci conferring seed coat color in soybean. Front Plant Sci 13:968618. 10.3389/fpls.2022.968618 35979081 10.3389/fpls.2022.968618 PMC9376438

[CR38] Zabala G, Vodkin LO (2014) Methylation affects transposition and splicing of a large CACTA transposon from a MYB transcription factor regulating anthocyanin synthase genes in soybean seed coats. PLoS ONE 9:e111959. 10.1371/journal.pone.0111959 25369033 10.1371/journal.pone.0111959 PMC4219821

[CR39] Zbinden-Foncea H, Castro-Sepulveda M, Fuentes J, Speisky H (2022) Effect of epicatechin on skeletal muscle: a review of its molecular mechanisms. Curr Med Chem 29:1110–1123. 10.2174/0929867328666210219114737 34923936 10.2174/0929867329666211217100020

