# Supplementary material for: Multivariate colorimetric phenotyping reveals genetic loci associated with soybean seed coat pigmentation and epicatechin accumulation
Source: Mol Breed. 2026 Mar 26;46(4):28. doi: 10.1007/s11032-026-01655-8 (PMC13022137; doi:10.1007/s11032-026-01655-8)

Table S1. L*, a*, b* and BLUP values in the parental lines and RILs derived from Jinpung x IT109098.

Trait	Year	Jinpung	IT109098	P-value[a]	RILs	H^2
L*	2020	54.27 ± 0.78	34.26 ± 0.45	***	43.76 ± 8.37	0.99
	2021	60.05 ± 1.59	29.59 ± 1.38	***	41.93 ± 10.42	0.98
BLUP		57.16 ± 3.15	31.93 ± 2.55	***	42.80 ± 8.40	0.90
a*	2020	0.46 ± 0.03	1.90 ± 0.34	**	1.46 ± 4.07	0.99
	2021	-0.96 ± 1.02	5.50 ± 1.82	*	4.17 ± 4.55	0.89
BLUP		-0.25 ± 1.01	3.71 ± 2.23	**	2.82 ± 4.02	0.88
b*	2020	26.22 ± 0.95	13.49 ± 1.00	***	18.82 ± 6.44	0.98
	2021	33.57 ± 1.19	14.71 ± 4.20	**	21.59 ± 6.72	0.79
BLUP		29.89 ± 3.83	14.09 ± 3.11	**	20.17 ± 5.87	0.80

[a] *: $P \leq 0.05$; **: $P \leq 0.01$; ***: $P \leq 0.001$

Table S2. Distribution and the mean of L*, a* and b* values according to groups of 'High-EC' and 'Low-EC'.

Trait	Group	Mean	Significance [a]	Range
L*	Low-EC	53.03 ± 4.83	***	36.55 ~ 65.66
	High- EC	32.76 ± 2.49		26.87 ~ 37.65
a*	Low-EC	2.10 ± 1.62	***	-0.64 ~ 8.31
	High- EC	5.47 ± 1.31		2.75 ~ 8.10
b*	Low-EC	26.81 ± 2.48	***	19.96 ~ 30.37
	High- EC	13.18 ± 2.66		6.37 ~ 18.07

[a] ***: $P \leq 0.001$

Table S3. Functional annotation of genes within the quantitative trait loci (QTLs).

Traits	QTL	Gene ID	*A.thaliana* ortholog	Gene symbol	Gene annotation	Description	Reference
**L*, PC1**	***qL6-1, qPC1-1***	Glyma.06G201600	AT5G07910		Leucine-rich repeat (LRR) family protein		
		Glyma.06G201700	AT1G32490	EMB2733,ESP3	RNA helicase family protein		
		Glyma.06G201800	AT5G07960				
		Glyma.06G201900					
		Glyma.06G202000	AT5G07980		dentin sialophosphoprotein-related		
		Glyma.06G202100	AT5G61150	VIP4	leo1-like family protein		
		Glyma.06G202200	AT1G74310	ATHSP101,HOT1,HSP101	heat shock protein 101		
		Glyma.06G202300	AT5G07990	CYP75B1,D501,TT7	Cytochrome P450 superfamily protein	Required for flavonoid 3' hydroxylase activity. Enzyme abundance relative to CHS determines Quercetin/Kaempferol metabolite ratio.	Knovich et al., 2011
		Glyma.06G202400	AT5G07990	CYP75B1,D501,TT7	Cytochrome P450 superfamily protein	Required for flavonoid 3' hydroxylase activity. Enzyme abundance relative to CHS determines Quercetin/Kaempferol metabolite ratio.	Knovich et al., 2011
		Glyma.06G202500	AT5G61140		U5 small nuclear ribonucleoprotein helicase		
		Glyma.06G202600	AT1G18650	PDCB3	plasmodesmata callose-binding protein 3		
		Glyma.06G202700					
		Glyma.06G202800					
		Glyma.06G202900	AT5G61090		Polynucleotidyl transferase, ribonuclease H-like superfamily protein		
		Glyma.06G203000	AT3G07820		Pectin lyase-like superfamily protein		
		Glyma.06G203100	AT5G61090		Polynucleotidyl transferase, ribonuclease H-like superfamily protein		
		Glyma.06G203200	AT2G33040	ATP3	gamma subunit of Mt ATP synthase		
		Glyma.06G203300	AT1G18640	PSP	3-phosphoserine phosphatase		

		Glyma.06G203400	AT1G25350	OVA9	glutamine-tRNA ligase, putative / glutaminyl-tRNA synthetase, putative / GlnRS, putative
		Glyma.06G203500			
		Glyma.06G203600	AT1G16820		vacuolar ATP synthase catalytic subunit-related / V-ATPase-related / vacuolar proton pump-related
		Glyma.06G203700	AT5G61040		
**L*, a*, b*,PC1**	***qEC08***	Glyma.08G114700	AT3G13600		calmodulin-binding family protein
		Glyma.08G114800	AT5G19830		Peptidyl-tRNA hydrolase family protein
		Glyma.08G114900	AT5G19820	emb2734	ARM repeat superfamily protein
		Glyma.08G115000			
		Glyma.08G115100	AT5G19780	TUA5	tubulin alpha-5
		Glyma.08G115200	AT2G18170	ATMPK7,MPK7	MAP kinase 7
		Glyma.08G115300	AT1G75390	AtbZIP44,bZIP44	basic leucine-zipper 44
		Glyma.08G115400	AT1G55320	AAE18	acyl-activating enzyme 18
		Glyma.08G115500	AT4G28590		
		Glyma.08G115600	AT5G19760		Mitochondrial substrate carrier family protein
		Glyma.08G115700	AT1G29120		Hydrolase-like protein family
		Glyma.08G115800	AT2G42590	GF14 MU,GRF9	general regulatory factor 9
		Glyma.08G115900	AT2G34140		Dof-type zinc finger DNA-binding family protein
		Glyma.08G116000	AT1G29170	ATSCAR3,SCAR3,WAVE2	SCAR family protein
		Glyma.08G116100	AT2G42610	LSH10	Protein of unknown function (DUF640)
		Glyma.08G116200	AT1G29195		
		Glyma.08G116300	AT1G09850	XBCP3	xylem bark cysteine peptidase 3
		Glyma.08G116400	AT1G09850	XBCP3	xylem bark cysteine peptidase 3
		Glyma.08G116500	AT4G18700	ATWL4,CIPK12,SnRK3.9,WL4	CBL-interacting protein kinase 12
		Glyma.08G116600	AT5G45840		Leucine-rich repeat protein kinase family protein
		Glyma.08G116700	AT2G38300		myb-like HTH transcriptional regulator family protein
		Glyma.08G116800	AT1G29240		Protein of unknown function (DUF688)
		Glyma.08G116900	AT5G43060		Granulin repeat cysteine protease family protein
		Glyma.08G117000	AT5G45800	MEE62	Leucine-rich repeat protein kinase family protein
		Glyma.08G117100	AT2G34190		Xanthine/uracil permease family protein

		Glyma.08G117200	AT4G18710	ATSK21,BIN2,DWF12,SK21,UCU1	Protein kinase superfamily protein
		Glyma.08G117300	AT2G34200		RING/FYVE/PHD zinc finger superfamily protein
		Glyma.08G117400	AT4G18750	DOT4	Pentatricopeptide repeat (PPR) superfamily protein
		Glyma.08G117500	AT4G18780	ATCESA8,CESA8,IRX1,LEW2	cellulose synthase family protein
		Glyma.08G117600	AT5G45760		Transducin/WD40 repeat-like superfamily protein
		Glyma.08G117700	AT3G30340		nodulin MtN21 /EamA-like transporter family protein
		Glyma.08G117800			
		Glyma.08G117900	AT3G19810		Protein of unknown function (DUF177)
		Glyma.08G118000	AT2G42760		
		Glyma.08G118100	AT1G29270		
		Glyma.08G118200	AT1G29280	ATWRKY65,WRKY65	WRKY DNA-binding protein 65
		Glyma.08G118300			
		Glyma.08G118400	AT4G18810		NAD(P)-binding Rossmann-fold superfamily protein
		Glyma.08G118500	AT1G29290		
		Glyma.08G118600	AT1G29300	UNE1	Plant protein of unknown function (DUF641)
		Glyma.08G118700	AT2G29420	ATGSTU7,GST25,GSTU7	glutathione S-transferase tau 7
		Glyma.08G118800	AT2G29420	ATGSTU7,GST25,GSTU7	glutathione S-transferase tau 7
		Glyma.08G118900	AT2G29420	ATGSTU7,GST25,GSTU7	glutathione S-transferase tau 7
		Glyma.08G119000	AT5G47260		ATP binding;GTP binding;nucleotide binding;nucleoside-triphosphatases
		Glyma.08G119100	AT3G09030		BTB/POZ domain-containing protein
		Glyma.08G119200	AT1G12280		LRR and NB-ARC domains-containing disease resistance protein
		Glyma.08G119300	AT1G29330	AERD2,ATERD2,ERD2	ER lumen protein retaining receptor family protein
		Glyma.08G119400	AT5G60490	FLA12	FASCICLIN-like arabinogalactan-protein 12
		Glyma.08G119500	AT5G60490	FLA12	FASCICLIN-like arabinogalactan-protein 12
		Glyma.08G119600	AT5G03170	ATFLA11,FLA11	FASCICLIN-like arabinogalactan-protein 11
**L***	***qL19-1***	Glyma.19G115400	AT5G14660	ATDEF2,DEF2,PDF1B	peptide deformylase 1B
		Glyma.19G115500			
		Glyma.19G115600	AT5G39890		Protein of unknown function (DUF1637)
		Glyma.19G115700	AT3G04480		endoribonucleases
		Glyma.19G115800	AT3G03770		Leucine-rich repeat protein kinase family protein

Glyma.19G115900	AT1G31220		Formyl transferase
Glyma.19G116000	AT5G35220	EGY1	Peptidase M50 family protein
Glyma.19G116100	AT1G28760		Uncharacterized conserved protein (DUF2215)
Glyma.19G116200	AT4G23620		Ribosomal protein L25/Gln-tRNA synthetase, anti-codon-binding domain
Glyma.19G116300	AT3G03740	ATBPM4,BPM4	BTB-POZ and MATH domain 4
Glyma.19G116400	AT1G58030	CAT2	cationic amino acid transporter 2
Glyma.19G116500	AT1G58030	CAT2	cationic amino acid transporter 2
Glyma.19G116600	AT1G80750		Ribosomal protein L30/L7 family protein
Glyma.19G116700	AT3G03710	PNP,RIF10	polyribonucleotide nucleotidyltransferase, putative
Glyma.19G116800	AT5G64200	ATSC35,SC35	ortholog of human splicing factor SC35
Glyma.19G116900			
Glyma.19G117000	AT2G44440		Emsy N Terminus (ENT) domain-containing protein
Glyma.19G117100	AT5G17860	CAX7	calcium exchanger 7
Glyma.19G117200	AT5G17850		Sodium/calcium exchanger family protein
Glyma.19G117300	AT5G17840		DnaJ/Hsp40 cysteine-rich domain superfamily protein
Glyma.19G117400	AT3G04440		Plasma-membrane choline transporter family protein
Glyma.19G117500			
Glyma.19G117600	AT3G03690	UNE7	Core-2/I-branching beta-1,6-N-acetylglucosaminyltransferase family protein
Glyma.19G117700	AT3G09360		Cyclin/Brf1-like TBP-binding protein
Glyma.19G117800			
Glyma.19G117900			
Glyma.19G118000	AT3G47500	CDF3	cycling DOF factor 3
Glyma.19G118100	AT4G26010		Peroxidase superfamily protein
Glyma.19G118200			
Glyma.19G118300			
Glyma.19G118400	AT3G03660	WOX11	WUSCHEL related homeobox 11

		Glyma.19G118500	AT1G26780	AtMYB117,LOF1,MYB117	myb domain protein 117	Encodes LOF1 (LATERAL ORGAN FUSION1), a MYB-domain transcription factor expressed in organ boundaries. Functions in boundary specification, meristem initiation and maintenance, and organ patterning	Ma et al., 2021
		Glyma.19G118600					
		Glyma.19G118700					
		Glyma.19G118800	AT4G03240	ATFH,FH	frataxin homolog		
		Glyma.19G118900	AT5G17790	VAR3	zinc finger (Ran-binding) family protein		
		Glyma.19G119000	AT5G17770	ATCBR,CBR,CBR1	NADH:cytochrome B5 reductase 1		
		Glyma.19G119100	AT3G45400		exostosin family protein		
		Glyma.19G119200	AT3G03630	CS26	cysteine synthase 26		
		Glyma.19G119300	AT1G08810	AtMYB60,MYB60	myb domain protein 60	putative transcription factor of the R2R3-MYB gene family. Transcript increases under conditions that promote stomatal opening (white and blue light, abi1-1 mutation) and decreases under conditions that trigger stomatal closure (ABA, desiccation, darkness), with the exception of elevated CO2. Expressed exclusively in guard cells of all tissues. It is required for light-induced opening of stomata. Mutant shows reduced stomatal aperture which helps to limit water loss during drought.	Park et al., 2008<br>Li et al., 2020
**a***	***qA1-1***	Glyma.01G182400	AT2G23450		Protein kinase superfamily protein		
		Glyma.01G182500	AT4G37200	HCF164	Thioredoxin superfamily protein		
		Glyma.01G182600	AT2G23670	YCF37	homolog of Synechocystis YCF37		
		Glyma.01G182700	AT4G37150	ATMES9,MES9	methyl esterase 9		
		Glyma.01G182800	AT5G41980				

Glyma.01G182900	AT2G24960		
Glyma.01G183000	AT5G65590		Dof-type zinc finger DNA-binding family protein
Glyma.01G183100	AT5G66920	sks17	SKU5 similar 17
Glyma.01G183200	AT2G23640	RTNLB13	Reticulan like protein B13
Glyma.01G183300	AT5G66900		Disease resistance protein (CC-NBS-LRR class) family
Glyma.01G183400	AT5G66900		Disease resistance protein (CC-NBS-LRR class) family
Glyma.01G183500	AT5G66880	SNRK2-3,SNRK2.3,SRK2I	sucrose nonfermenting 1(SNF1)-related protein kinase 2.3
Glyma.01G183600	AT3G19080		SWIB complex BAF60b domain-containing protein
Glyma.01G183700	AT4G37180		Homeodomain-like superfamily protein
Glyma.01G183800	AT4G37190		
Glyma.01G183900	AT5G66870	ASL1,LBD36	ASYMMETRIC LEAVES 2-like 1
Glyma.01G184000	AT5G66850	MAPKKK5	mitogen-activated protein kinase kinase kinase 5
Glyma.01G184100			
Glyma.01G184200	AT1G49570		Peroxidase superfamily protein
Glyma.01G184300	AT3G50530	CRK	CDPK-related kinase
Glyma.01G184400	AT5G66840		SAP domain-containing protein
Glyma.01G184500	AT3G50590		Transducin/WD40 repeat-like superfamily protein
Glyma.01G184600	AT4G37080		Protein of unknown function, DUF547
Glyma.01G184700	AT5G66820		
Glyma.01G184800			
Glyma.01G184900	AT2G23440		
Glyma.01G185000	AT3G50610		
Glyma.01G185100	AT5G66815		
Glyma.01G185200	AT3G50620		P-loop containing nucleoside triphosphate hydrolases superfamily protein
Glyma.01G185300	AT4G37040	MAP1D	methionine aminopeptidase 1D
Glyma.01G185400	AT1G49620	ICK5,ICN6,KRP7	Cyclin-dependent kinase inhibitor family protein
Glyma.01G185500	AT5G66800		
Glyma.01G185600	AT2G15680		Calcium-binding EF-hand family protein
Glyma.01G185700	AT4G37020		
Glyma.01G185800	AT4G36990	AT-HSFB1,ATHSF4,HSF4,HSFB1	heat shock factor 4

Glyma.01G185900	AT4G33985		Protein of unknown function (DUF1685)
Glyma.01G186000	AT4G36980		
Glyma.01G186100	AT5G67020		
Glyma.01G186200	AT5G67030	ABA1,ATABA1,ATZEP,IBS3,LOS6,NPQ2,ZEP	zeaxanthin epoxidase (ZEP) (ABA1)
Glyma.01G186300	AT1G45207		Remorin family protein
Glyma.01G186400	AT4G36960		RNA-binding (RRM/RBD/RNP motifs) family protein
Glyma.01G186500			
Glyma.01G186600	AT5G42930		alpha/beta-Hydrolases superfamily protein
Glyma.01G186700	AT5G67060	HEC1	basic helix-loop-helix (bHLH) DNA-binding superfamily protein
Glyma.01G186800	AT5G67070	RALFL34	ralf-like 34
Glyma.01G186900	AT4G36950	MAPKKK21	mitogen-activated protein kinase kinase kinase 21
Glyma.01G187000	AT2G24670		Domain of unknown function (DUF313)
Glyma.01G187100	AT2G24670		Domain of unknown function (DUF313)
Glyma.01G187200	AT4G36945		PLC-like phosphodiesterases superfamily protein
Glyma.01G187300	AT4G02630		Protein kinase superfamily protein
Glyma.01G187400	AT5G67100	ICU2	DNA-directed DNA polymerases
Glyma.01G187500	AT2G23420	NAPRT2	nicotinate phosphoribosyltransferase 2
Glyma.01G187600	AT4G36930	SPT	basic helix-loop-helix (bHLH) DNA-binding superfamily protein
Glyma.01G187700	AT4G34050	CCoAOMT1	S-adenosyl-L-methionine-dependent methyltransferases superfamily protein
Glyma.01G187800	AT5G67140		F-box/RNI-like superfamily protein
Glyma.01G187900	AT5G67130		PLC-like phosphodiesterases superfamily protein
Glyma.01G188000	AT2G23380	CLF,ICU1,SDG1,SET1	SET domain-containing protein
Glyma.01G188100	AT4G34090		
Glyma.01G188200	AT2G23360		Plant protein of unknown function (DUF869)
Glyma.01G188300			
Glyma.01G188400	AT4G36920	AP2,FL1,FLO2	Integrase-type DNA-binding superfamily protein
Glyma.01G188500	AT4G36910	CDCP2,LEJ2	Cystathionine beta-synthase (CBS) family protein
Glyma.01G188600	AT5G67190	DEAR2	DREB and EAR motif protein 2
Glyma.01G188700	AT2G42240		RNA-binding (RRM/RBD/RNP motifs) family protein

Glyma.01G188800	AT2G36780		UDP-Glycosyltransferase superfamily protein
Glyma.01G188900	AT3G57870	AHUS5,ATSCE1,EMB1637,SCE1,SCE1A	sumo conjugation enzyme 1
Glyma.01G189000	AT5G67210		Protein of unknown function (DUF579)
Glyma.01G189100	AT2G23320	WRKY15	WRKY DNA-binding protein 15
Glyma.01G189200	AT5G67230	I14H,IRX14-L	Nucleotide-diphospho-sugar transferases superfamily protein
Glyma.01G189300	AT3G50120		Plant protein of unknown function (DUF247)
Glyma.01G189400	AT4G39220	ATRER1A	Rer1 family protein
Glyma.01G189500	AT3G19420	ATPEN2,PEN2	PTEN 2
Glyma.01G189600	AT5G67250	SKIP2,VFB4	SKP1/ASK1-interacting protein 2
Glyma.01G189700	AT5G67260	CYCD3;2	CYCLIN D3;2
Glyma.01G189800	AT3G62290	ARFA1E,ATARFA1E	ADP-ribosylation factor A1E
Glyma.01G189900	AT5G67265		
Glyma.01G190000	AT5G67270	ATEB1C,ATEB1H1,EB1C	end binding protein 1C
Glyma.01G190100	AT4G37260	ATMYB73,MYB73	myb domain protein 73
Glyma.01G190200			
Glyma.01G190300	AT4G37300	MEE59	maternal effect embryo arrest 59
Glyma.01G190400	AT4G37330	CYP81D4	cytochrome P450, family 81, subfamily D, polypeptide 4
Glyma.01G190500	AT2G15000		
Glyma.01G190600	AT2G14960	GH3.1	Auxin-responsive GH3 family protein
Glyma.01G190700	AT2G23150	ATNRAMP3,NRAMP3	natural resistance-associated macrophage protein 3
Glyma.01G190800	AT5G43100		Eukaryotic aspartyl protease family protein
Glyma.01G190900	AT4G37450	AGP18,ATAGP18	arabinogalactan protein 18
Glyma.01G191000	AT5G43120		ARM-repeat/Tetratricopeptide repeat (TPR)-like protein
Glyma.01G191100	AT4G37460	SRFR1	Tetratricopeptide repeat (TPR)-like superfamily protein
Glyma.01G191200	AT4G37470		alpha/beta-Hydrolases superfamily protein
Glyma.01G191300	AT4G33920		Protein phosphatase 2C family protein
Glyma.01G191400	AT5G67350		
Glyma.01G191500	AT4G37480		Chaperone DnaJ-domain superfamily protein
Glyma.01G191600	AT5G67370		Protein of unknown function (DUF1230)
Glyma.01G191700	AT4G33910		2-oxoglutarate (2OG) and Fe(II)-dependent oxygenase superfamily protein

		Glyma.01G191800	AT2G23093		Major facilitator superfamily protein
		Glyma.01G191900	AT4G24230	ACBP3	acyl-CoA-binding domain 3
		Glyma.01G192000	AT2G23090		Uncharacterised protein family SERF
		Glyma.01G192100	AT5G67380	ATCKA1,CKA1	casein kinase alpha 1
**a*, PC2**	***qEC01***	Glyma.01G198900	AT4G17950		AT hook motif DNA-binding family protein
		Glyma.01G199000	AT5G46630		Clathrin adaptor complexes medium subunit family protein
		Glyma.01G199100	AT4G17960		
		Glyma.01G199200	AT1G32540	LOL1	lsd one like 1
		Glyma.01G199300			
		Glyma.01G199400	AT4G17970	ALMT12,ATALMT12	aluminum-activated, malate transporter 12
		Glyma.01G199500	AT1G32500	ATNAP6,NAP6	non-intrinsic ABC protein 6
		Glyma.01G199600	AT2G35350	PLL1	poltergeist like 1
		Glyma.01G199700	AT2G35370	GDCH	glycine decarboxylase complex H
		Glyma.01G199800	AT2G32440	ATKAO2,CYP88A4,KAO2	ent-kaurenoic acid hydroxylase 2
		Glyma.01G199900	AT1G32460		
		Glyma.01G200000	AT5G64816		
		Glyma.01G200100	AT1G32450	NRT1.5	nitrate transporter 1.5
		Glyma.01G200200	AT1G32440	PKp3	plastidial pyruvate kinase 3
		Glyma.01G200300			
		Glyma.01G200400	AT2G35380		Peroxidase superfamily protein
		Glyma.01G200500	AT4G18010	5PTASE2,AT5PTASE2,IP5PII	myo-inositol polyphosphate 5-phosphatase 2
		Glyma.01G200600	AT5G04820	ATOFP13,OFP13	ovate family protein 13
		Glyma.01G200700	AT1G32400	TOM2A	tobamovirus multiplication 2A
		Glyma.01G200800	AT4G18020	APRR2,PRR2	CheY-like two-component responsive regulator family protein
		Glyma.01G200900	AT5G46560		
		Glyma.01G201000	AT1G32990	PRPL11	plastid ribosomal protein l11
		Glyma.01G201100	AT1G32410		Vacuolar protein sorting 55 (VPS55) family protein
		Glyma.01G201200	AT3G59840		
		Glyma.01G201300	AT5G18070	DRT101	phosphoglucosamine mutase-related
		Glyma.01G201400	AT5G18070	DRT101	phosphoglucosamine mutase-related
		Glyma.01G201500	ATCG00890		NADH-Ubiquinone/plastoquinone (complex I) protein

Glyma.01G201600	ATCG00360		Tetratricopeptide repeat (TPR)-like superfamily protein
Glyma.01G201700	ATCG00660		ribosomal protein L20
Glyma.01G201800	AT1G32415		pentatricopeptide (PPR) repeat-containing protein
Glyma.01G201900	AT2G38740		Haloacid dehalogenase-like hydrolase (HAD) superfamily protein
Glyma.01G202000	AT2G38740		Haloacid dehalogenase-like hydrolase (HAD) superfamily protein
Glyma.01G202100	AT4G17030	AT-EXPR,ATEXLB1,ATEXPR1,ATHEXP BETA 3.1,EXLB1,EXPR	expansin-like B1
Glyma.01G202200	AT2G45760	BAL,BAP2	BON association protein 2
Glyma.01G202300	AT1G07615		GTP-binding protein Obg/CgtA
Glyma.01G202400	AT2G44610	ATRAB6A,ATRABH1B,RAB6,RAB6A	Ras-related small GTP-binding family protein
Glyma.01G202500	AT2G44610	ATRAB6A,ATRABH1B,RAB6,RAB6A	Ras-related small GTP-binding family protein
Glyma.01G202600	AT5G47010	ATUPF1,LBA1,UPF1	RNA helicase, putative
Glyma.01G202700	AT2G22740	SDG23,SUVH6	SU(VAR)3-9 homolog 6
Glyma.01G202800	AT4G38180	FRS5	FAR1-related sequence 5
Glyma.01G202900	AT1G10240	FRS11	FAR1-related sequence 11
Glyma.01G203000	AT5G47020		
Glyma.01G203100	AT5G47040	LON2	lon protease 2
Glyma.01G203200	AT5G47060		Protein of unknown function (DUF581)
Glyma.01G203300	AT1G32740		SBP (S-ribonuclease binding protein) family protein
Glyma.01G203400	AT4G17090	BAM3,BMY8,CT-BMY	chloroplast beta-amylase
Glyma.01G203500	AT5G47070		Protein kinase superfamily protein
Glyma.01G203600	AT3G10950		Zinc-binding ribosomal protein family protein
Glyma.01G203700			
Glyma.01G203800	AT4G17640	CKB2	casein kinase II beta chain 2
Glyma.01G203900	AT4G17620		glycine-rich protein
Glyma.01G204000	AT4G17620		glycine-rich protein
Glyma.01G204100	AT5G47100	ATCBL9,CBL9	calcineurin B-like protein 9

		Glyma.01G204200	AT4G33950	ATOST1,OST1,P44,SNRK2-6,SNRK2.6,SRK2E	Protein kinase superfamily protein
		Glyma.01G204300	AT4G17070		peptidyl-prolyl cis-trans isomerases
		Glyma.01G204400	AT1G74950	JAZ2,TIFY10B	TIFY domain/Divergent CCT motif family protein
		Glyma.01G204500	AT5G41150	ATRAD1,RAD1,UVH1	Restriction endonuclease, type II-like superfamily protein
		Glyma.01G204600	AT5G07440	GDH2	glutamate dehydrogenase 2
		Glyma.01G204700	AT4G17600	LIL3:1	Chlorophyll A-B binding family protein
		Glyma.01G204800	AT4G17030	AT-EXPR,ATEXLB1,ATEXPR1,ATHEXP BETA 3.1,EXLB1,EXPR	expansin-like B1
		Glyma.01G204900	AT2G22740	SDG23,SUVH6	SU(VAR)3-9 homolog 6
		Glyma.01G205000	AT1G78010		tRNA modification GTPase, putative
		Glyma.01G205100	AT4G17570	GATA26	GATA transcription factor 26
		Glyma.01G205200	AT5G47120	ATBI-1,ATBI1,BI-1,BI1	BAX inhibitor 1
		Glyma.01G205300	AT5G47180		Plant VAMP (vesicle-associated membrane protein) family protein
		Glyma.01G205400	AT4G17560		Ribosomal protein L19 family protein
		Glyma.01G205500	AT4G17540		
		Glyma.01G205600	AT4G17540		
**a***	***qA5-1***	Glyma.05G042100			
		Glyma.05G042200	AT5G50200	ATNRT3.1,NRT3.1,WR3	nitrate transmembrane transporters
		Glyma.05G042300	AT4G24730		Calcineurin-like metallo-phosphoesterase superfamily protein
		Glyma.05G042400	AT5G50210	OLD5,QS,SUFE3	quinolinate synthase
		Glyma.05G042500	AT4G13310	CYP71A20	cytochrome P450, family 71, subfamily A, polypeptide 20
		Glyma.05G042600	AT4G13310	CYP71A20	cytochrome P450, family 71, subfamily A, polypeptide 20
		Glyma.05G042700	AT4G24740	AFC2,AME1,FC2	FUS3-complementing gene 2
		Glyma.05G042800	AT1G13110	CYP71B7	cytochrome P450, family 71 subfamily B, polypeptide 7
		Glyma.05G042900	AT5G14410		
		Glyma.05G043000	AT3G55740	ATPROT2,PROT2	proline transporter 2
		Glyma.05G043100	AT2G39890	ATPROT1,PROT1	proline transporter 1
		Glyma.05G043200	AT5G50250	CP31B	chloroplast RNA-binding protein 31B

		Glyma.05G043300	AT3G48330	ATPIMT1,PIMT1	protein-l-isoaspartate methyltransferase 1
		Glyma.05G043400	AT1G78790		
		Glyma.05G043500	AT1G24530		Transducin/WD40 repeat-like superfamily protein
		Glyma.05G043600	AT4G24760		alpha/beta-Hydrolases superfamily protein
		Glyma.05G043700	AT5G50230		Transducin/WD40 repeat-like superfamily protein
		Glyma.05G043800			
		Glyma.05G043900	AT3G48360	ATBT2,BT2	BTB and TAZ domain protein 2
		Glyma.05G044000	AT5G63180		Pectin lyase-like superfamily protein
		Glyma.05G044100	AT4G24790		AAA-type ATPase family protein
		Glyma.05G044200	AT5G63190		MA3 domain-containing protein
		Glyma.05G044300	AT3G48440		Zinc finger C-x8-C-x5-C-x3-H type family protein
		Glyma.05G044400	AT5G63270		RPM1-interacting protein 4 (RIN4) family protein
		Glyma.05G044500	AT4G24820		26S proteasome, regulatory subunit Rpn7;Proteasome component (PCI) domain
		Glyma.05G044600	AT3G48460		GDSL-like Lipase/Acylhydrolase superfamily protein
		Glyma.05G044700	AT1G24440		RING/U-box superfamily protein
		Glyma.05G044800	AT3G27160	GHS1	Ribosomal protein S21 family protein
		Glyma.05G044900	AT4G24820		26S proteasome, regulatory subunit Rpn7;Proteasome component (PCI) domain
		Glyma.05G045000	AT5G50330		Protein kinase superfamily protein
		Glyma.05G045100	AT3G48490		
		Glyma.05G045200	AT5G63310	ATNDPK2,NDPK IA,NDPK IA IA,NDPK1A,NDPK2	nucleoside diphosphate kinase 2
		Glyma.05G045300	AT1G66350	RGL,RGL1	RGA-like 1
		Glyma.05G045400	AT5G50335		
		Glyma.05G045500	AT5G09840		Putative endonuclease or glycosyl hydrolase
		Glyma.05G045600	AT1G24400	AATL2,ATLHT2,LHT2	lysine histidine transporter 2
		Glyma.05G045700	AT5G63320	NPX1	nuclear protein X1
		Glyma.05G045800	AT5G63350		
		Glyma.05G045900	AT5G03860	MLS	malate synthase
**b***	***qB6-1***	Glyma.06G207100	AT1G73990	SPPA,SPPA1	signal peptide peptidase
		Glyma.06G207200	AT1G73990	SPPA,SPPA1	signal peptide peptidase
		Glyma.06G207300	AT3G07840		Pectin lyase-like superfamily protein

Glyma.06G207400	AT1G09690		Translation protein SH3-like family protein
Glyma.06G207500	AT3G14920		Peptide-N4-(N-acetyl-beta-glucosaminyl)asparagine amidase A protein
Glyma.06G207600	AT5G38790		
Glyma.06G207700	AT3G52140		tetratricopeptide repeat (TPR)-containing protein
Glyma.06G207800	AT2G33720		AP2/B3-like transcriptional factor family protein
Glyma.06G207900	AT1G27090		glycine-rich protein
Glyma.06G208000	AT1G74000	SS3	strictosidine synthase 3
Glyma.06G208100	AT1G18490		Protein of unknown function (DUF1637)
Glyma.06G208200	AT1G74030	ENO1	enolase 1
Glyma.06G208300	AT1G18520	TET11	tetraspanin11
Glyma.06G208400	AT5G23050	AAE17	acyl-activating enzyme 17
Glyma.06G208500			
Glyma.06G208600			
Glyma.06G208700	AT1G71900		Protein of unknown function (DUF803)
Glyma.06G208800	AT1G18530		EF hand calcium-binding protein family
Glyma.06G208900	AT1G68710		ATPase E1-E2 type family protein / haloacid dehalogenase-like hydrolase family protein
Glyma.06G209000	AT5G23090	NF-YB13	nuclear factor Y, subunit B13
Glyma.06G209100	AT5G23100		Protein of unknown function, DUF617
Glyma.06G209200	AT1G18550		ATP binding microtubule motor family protein
Glyma.06G209300	AT1G05230	HDG2	homeodomain GLABROUS 2
Glyma.06G209400	AT3G18600		P-loop containing nucleoside triphosphate hydrolases superfamily protein
Glyma.06G209500	AT3G18490		Eukaryotic aspartyl protease family protein
Glyma.06G209600	AT1G48960		Adenine nucleotide alpha hydrolases-like superfamily protein
Glyma.06G209700	AT5G38640		NagB/RpiA/CoA transferase-like superfamily protein
Glyma.06G209800	AT3G25690	CHUP1	Hydroxyproline-rich glycoprotein family protein
Glyma.06G209900	AT3G18570		Oleosin family protein
Glyma.06G210000	AT5G41220	ATGSTT3,GST10C,GSTT3	glutathione S-transferase THETA 3
Glyma.06G210200	AT1G49000		
Glyma.06G210300			

		Glyma.06G210400	AT1G03280		Transcription factor TFIIE, alpha subunit
		Glyma.06G210500			
		Glyma.06G210600	AT3G18550	ATTCP18,BRC1,TCP18	TCP family transcription factor
		Glyma.06G210700	AT1G68310		Protein of unknown function (DUF59)
		Glyma.06G210800	AT1G73930		
		Glyma.06G210900	AT3G24330		O-Glycosyl hydrolases family 17 protein
		Glyma.06G211000	AT4G02210		
		Glyma.06G211100	AT3G51690		PIF1 helicase
		Glyma.06G211200	AT1G03390		HXXXD-type acyl-transferase family protein
		Glyma.06G211300	AT1G08510	FATB	fatty acyl-ACP thioesterases B
		Glyma.06G211400	AT5G22950	VPS24.1	SNF7 family protein
		Glyma.06G211500	AT5G19950		Domain of unknown function (DUF1767)
		Glyma.06G211600	AT2G26730		Leucine-rich repeat protein kinase family protein
		Glyma.06G211700			
		Glyma.06G211800			
		Glyma.06G211900	AT5G55740	CRR21	Tetratricopeptide repeat (TPR)-like superfamily protein
		Glyma.06G212000			
**PC2**	***qPC2-2***	Glyma.06G203800	AT5G61030	GR-RBP3	glycine-rich RNA-binding protein 3
		Glyma.06G203900	AT5G61010	ATEXO70E2,EXO70E2	exocyst subunit exo70 family protein E2
		Glyma.06G204000	AT1G74220		
		Glyma.06G204100	AT3G29575	AFP3	ABI five binding protein 3
		Glyma.06G204200	AT5G08050		Protein of unknown function (DUF1118)
		Glyma.06G204300	AT5G60970	TCP5	TEOSINTE BRANCHED 1, cycloidea and PCF transcription factor 5
		Glyma.06G204400	AT1G74160		
		Glyma.06G204500	AT4G26190		Haloacid dehalogenase-like hydrolase (HAD) superfamily protein
		Glyma.06G204600	AT3G26850		histone-lysine N-methyltransferases

Table S4. Variations in sequence between IT109098 and Jinpung in QTL regions.

Trait	QTL	Gene ID	Number of variants			Predicted change
			-2kb Upstream	Genic region	+2kb Downstream	(reference: Jinpung)[a]
**L***	***qL19-1***	Glyma.19G115400	18	2	11	p.(Ala52Ala), p.(Ser9Pro)
		Glyma.19G115500	17	1	31	p.(Ser20Ser)
		Glyma.19G115600	34		15	
		Glyma.19G115700	20	4	19	start_lost, p.(Asn365Lys), p.(Gly449Gly), p.(Thr606Ile)
		Glyma.19G115800	16	1	11	p.(Pro737Pro)
		Glyma.19G115900	25	6	54	p.(Val97Ile), p.(Ser78_Ser79del), p.(Cys72Trp), p.(Phe57Ser), p.(Pro53Pro), p.(Pro43Thr)
		Glyma.19G116000	66		41	
		Glyma.19G116100	41	1		p.(Leu148Ser)
		Glyma.19G116200	1		3	
		Glyma.19G116300	59		1	
		Glyma.19G116400	18	1	46	p.(Ala439Val)
		Glyma.19G116500	46	4	22	p.(Lys39Arg), p.(Pro273Pro), p.(Val294Leu), p.(Thr513Ala)
		Glyma.19G116600	36		12	
		Glyma.19G116700	11		17	
		Glyma.19G116800	11	4	13	p.(Val359Ile), p.(Val322Ala), p.(Met160Thr), frameshift p.(Arg104fs)
		Glyma.19G116900	12	1	19	frameshift p.(Glu13fs)
		Glyma.19G117000	11		9	
		Glyma.19G117200	13		3	
		Glyma.19G117300	31	1	2	p.(Ser106Pro)
		Glyma.19G117400	13	1	41	p.(Arg123Lys)
		Glyma.19G117500	13		16	p.(Glu50Val)
		Glyma.19G117600	28	4	3	p.(Met72Leu), p.(Lys73Arg), p.(Phe392Ile), p.(Phe392Tyr)
		Glyma.19G117700	24	2	31	p.(Phe346Val), p.(Val386Phe)

		Glyma.19G117800	16		1	
		Glyma.19G117900	7		6	
		Glyma.19G118000	16	3	12	p.(Met211Val), p.(Ala319Ser), p.(Ile450Asn)
		Glyma.19G118100	24	1	13	p.(Arg38Gly)
		Glyma.19G118200	16	1	14	p.(Thr47Ile)
		Glyma.19G118300	6		17	
		Glyma.19G118400	18	2	20	p.(Ala101Thr), p.(Cys144Ser)
		Glyma.19G118500	15		1	
		Glyma.19G118700	1			
		Glyma.19G118800	5		3	
		Glyma.19G118900	2		5	
		Glyma.19G119000	8	1	20	p.(Tyr30Asn)
		Glyma.19G119100	20	1	28	p.(Met508Lys)
		Glyma.19G119200	49	2	25	start_lost p.(Met1?), inframe_insertion p.(Gln29_Leu30insArgHisHis)
		Glyma.19G119300		1	6	p.(Ser295Phe)
**a***	***qA1-1***	Glyma.01G182400	20	1	16	p.(Gly253Arg)
		Glyma.01G182500	16		8	
		Glyma.01G182600	47		21	
		Glyma.01G182700	23		6	p.(Gly22Arg)
		Glyma.01G182800	34	14	34	p.(Lys218Glu), p.(Lys212Glu), p.(Pro190Leu), p.(Thr177Met), p.(Asp114Gly), p.(Ser98Gly), p.(Cys93Tyr), p.(Gln73Arg), p.(Arg66Cys), p.(Ser49Asn), p.(Ser12Pro)
		Glyma.01G182900	59	10	34	p.(Pro182Leu), p.(Phe172Ser), p.(Ser144Ala), p.(Thr138Ile), p.(Ser137Leu), p.(Gly129Ser), p.(Glu105Gly), p.(Val96Leu), p.(Glu12Ala)
		Glyma.01G183000	20		9	
		Glyma.01G183100	18		20	
		Glyma.01G183200	19		6	
		Glyma.01G183300	6		26	

Glyma.01G183400	26	1	3	p.(Ala708Val)
Glyma.01G183500	4		5	
Glyma.01G183600	8		8	
Glyma.01G183700	55		11	p.(Ala176Thr)
Glyma.01G183800	13		20	
Glyma.01G183900	2		22	
Glyma.01G184300	2			
Glyma.01G185000	1			
Glyma.01G185100			1	
Glyma.01G185200			1	
Glyma.01G185400			1	
Glyma.01G185700			1	
Glyma.01G186700	6	1	16	insertion p.(Asn38_Thr39insThrThrThr)
Glyma.01G186800	1		14	
Glyma.01G186900	9			
Glyma.01G187000			5	
Glyma.01G187100	3		20	
Glyma.01G187200	10	2	5	p.(Ala46Thr), p.(Thr377Ser)
Glyma.01G187300	27	1	26	frameshift p.(Asp464fs)
Glyma.01G187400	20	6	9	p.(Val225Glu), p.(Glu279Ala), p.(Ile290Lys), p.(Val646Ala), p.(His669Tyr), p.(Leu1333Phe)
Glyma.01G187500	17		6	
Glyma.01G187600	33	2	18	p.(Lys239Asn), p.(Pro88Ser),
Glyma.01G187700	4		13	
Glyma.01G187800	5			
Glyma.01G187900	2			
Glyma.01G188000	6		2	

Glyma.01G188100	14		11	
Glyma.01G188200	13	8	10	p.(Lys649Arg), p.(Phe581Tyr), p.(Pro572Ser), p.(Thr506Ile), p.(Pro482Ala), p.(Gln426Arg), p.(Thr395Lys), p.(Ile207Thr),
Glyma.01G188300	27	1	16	p.(Leu126Val)
Glyma.01G188400	35		4	
Glyma.01G188500	1		9	
Glyma.01G188600	39	1	4	p.(Gly139Asp)
Glyma.01G188700	1	1	1	deletion p.(Pro24_Leu26del)
Glyma.01G188900	1			
Glyma.01G189400	1			
Glyma.01G189700	1			
Glyma.01G189800	1			
Glyma.01G189900			1	
Glyma.01G190000	1		1	
Glyma.01G190100	4	1	19	insertion p.(Pro226_Leu227insGlnPro)
Glyma.01G190200	5		24	
Glyma.01G190300	13		5	
Glyma.01G190400	5	1	2	p.(Ser59Leu)
Glyma.01G190500	1			
Glyma.01G190600		1	7	p.(Ser431Ala)
Glyma.01G190700	31	1	8	p.(Gly430Asp)
Glyma.01G190800	13	1	16	p.(Asp72His)
Glyma.01G190900	20	1	13	p.(Ser27Ala)
Glyma.01G191000	1	2	8	p.(Pro288Ser), p.(Thr310Met)
Glyma.01G191100	16	1	7	p.(Arg752Cys)
Glyma.01G191200	6	1	36	p.(Ser270Ala)
Glyma.01G191300	10	1	11	p.(Cys12Trp)

		Glyma.01G191400	10	5	13	p.(Gln245His), p.(Glu64Asp), p.(Gln57Arg), p.(Ala29Glu), deletion p.(Glu17_Glu19del)
		Glyma.01G191500	38		2	
		Glyma.01G191600	22		7	
		Glyma.01G191700	11	2	54	p.(Val105Ala), frameshift p.(Asp212fs),
		Glyma.01G191800	19	1	84	p.(Thr275Ile)
		Glyma.01G191900	20	1	105	p.(Asn210Tyr)
		Glyma.01G192000	18		10	
		Glyma.01G192100			11	
a*, PC2	***qEC01***	Glyma.01G198900	7			
		Glyma.01G199000	11	4	2	earlystop p.(Gln7*), earlystop p.(Gln126*), p.(Ser82Cys), p.(Ser201Cys)
		Glyma.01G199100	2		13	
		Glyma.01G199200	1		12	
		Glyma.01G199300		1	2	frameshift p.(Ser21fs)
		Glyma.01G199400	12		18	
		Glyma.01G199500	11	8	28	frameshift p.(Leu10fs), p.(Ala65Thr), p.(Ala81Thr), p.(His130Leu), p.(Phe136Ile), p.(Gly182Val), p.(Asp219Tyr), p.(Gln255Leu)
		Glyma.01G199600	29	6	52	earlystop p.(Cys588*), p.(Asp554Glu), p.(Gly438Glu), p.(Ser295Pro), p.(Leu159Val), p.(Ala158Thr)
		Glyma.01G199700	17	3	13	p.(Cys7Tyr), p.(Thr53Ala), p.(Thr154Thr)
		Glyma.01G199800	6	1	9	p.(Pro59Leu)
		Glyma.01G199900	8		6	
		Glyma.01G200000	8		5	
		Glyma.01G200100	16		7	
		Glyma.01G200200		3	13	p.(Val496Ala), p.(Phe189Val), p.(Pro60His)
		Glyma.01G200300	1		1	
		Glyma.01G200400	7		1	
		Glyma.01G200500	2		7	

		Glyma.01G200600	24	1	33	p.(Ala25Val)
		Glyma.01G200700			3	
		Glyma.01G201400		1		p.(Phe282Leu)
		Glyma.01G201500	1			
		Glyma.01G202300	1			
		Glyma.01G202400			1	
		Glyma.01G202600	1			
		Glyma.01G203000			1	
		Glyma.01G203100	1		1	
		Glyma.01G203600			1	
		Glyma.01G203700	1			
		Glyma.01G203800	1			
		Glyma.01G203900	1		1	
		Glyma.01G204200			1	
		Glyma.01G204300		1		p.(Asp109Asn)
		Glyma.01G205100	1			
		Glyma.01G205200	1		1	
		Glyma.01G205400	1			
		Glyma.01G205600			1	
**a***	***qA5-1***	Glyma.05G042100	21	7		p.(Val363Ala), p.(Val222Ala), p.(Arg142Gln), p.(Arg283Gln), start_lost p.(Met1?), insertion p.(His60delinsProValAsp)
		Glyma.05G042200	5		7	
		Glyma.05G042300	11	1	9	p.(Asn207Ser)
		Glyma.05G042400	11		8	
		Glyma.05G042500	12		1	
		Glyma.05G042600	1			
		Glyma.05G042700			1	

		Glyma.05G043400	2			
		Glyma.05G043500	1		1	
		Glyma.05G043600	12		5	
		Glyma.05G043700	6	1	8	p.(Gly5Trp)
		Glyma.05G043800	16		9	
		Glyma.05G043900	5	1	23	frameshift p.(Met196fs)
		Glyma.05G044100	18	4		p.(Lys351Gln), p.(Lys396Gln), p.(Lys430Glu), p.(Lys475Glu)
		Glyma.05G044200	13		1	
		Glyma.05G044300	6	1	1	p.(Val9Ala)
		Glyma.05G044400	6		2	
		Glyma.05G044500	8		36	
		Glyma.05G044600	4		1	
		Glyma.05G045200	1		1	
		Glyma.05G045300	1	1		p.(Glu304Lys)
		Glyma.05G045500	92	1	3	p.(Arg155Ile)
		Glyma.05G045600	9		8	
		Glyma.05G045700	30	2	8	p.(Leu388Arg), p.(Leu35Ser)
		Glyma.05G045800	18	1	5	p.(Cys77Arg)
		Glyma.05G045900	17			
**b***	***qB6-1***	Glyma.06G207200	7	3	6	p.(Pro86Ser), p.(Val61Ala), p.(Asn3Lys)
		Glyma.06G207300	2	1	2	p.(His289Tyr)
		Glyma.06G207400	24	3	9	p.(Ser2Trp), p.(Thr24Ala), p.(Ala58Val)
		Glyma.06G207500	17	1	3	p.(Lys32Arg)
		Glyma.06G207600	28		7	
		Glyma.06G207700	1	1		p.(Arg138His)
		Glyma.06G207800	3	1	3	frameshift p.(Asn22fs)

Glyma.06G207900	45	7	93	p.(Pro75Gln), p.(Leu73Pro), p.(Ala42Thr), p.(Ser29Ile), p.(Gln15His), p.(Ile12Asn), p.(Asp3Glu)
Glyma.06G208000	41	1	8	p.(Leu2Trp)
Glyma.06G208100	6		13	
Glyma.06G208200	46		23	
Glyma.06G208300	18	6	23	p.(Val8Ala), p.(Ile45Val), p.(Ser50Gly), p.(Pro85Leu), p.(Ala161Val), p.(Lys192Glu)
Glyma.06G208400	11	1	14	p.(Pro57His)
Glyma.06G208500	10		12	
Glyma.06G208600	7		13	
Glyma.06G208700	13	1	18	p.(Asn89Ser)
Glyma.06G208800	14		28	
Glyma.06G208900	27	4	20	p.(Ile203Met), p.(Ile304Met), p.(Cys127Gly), p.(Cys228Gly)
Glyma.06G209000	26		34	
Glyma.06G209100	31		5	
Glyma.06G209200	6		6	
Glyma.06G209300	13	2	5	p.(Glu227Lys), p.(Lys18Asn)
Glyma.06G209400	16	2	3	p.(Ala416Ser), p.(Ser527Cys)
Glyma.06G209500	5	3	3	p.(Glu304Val), p.(Met270Val), p.(Ala28Glu)
Glyma.06G209600	11		9	
Glyma.06G209700	7	1	10	p.(Lys262Ile)
Glyma.06G209800	4	1	2	p.(Met235Ile)
Glyma.06G209900	6		7	
Glyma.06G210000	3	4	3	p.(Arg235His), p.(Ser218Pro), p.(Gln192Lys), p.(Gly168Asp)
Glyma.06G210100	5	2	3	p.(Thr487Ile), p.(Arg426Lys)
Glyma.06G210200	23		3	
Glyma.06G210300	11	2	13	p.(Leu129Phe), p.(Val74Met)
Glyma.06G210400	27		17	

		Glyma.06G210500	20	2	17	p.(Ala41Val), p.(Asp44Tyr)
		Glyma.06G210600	38	3	3	p.(Glu295Ala), frameshift p.(Phe156fs), p.(Phe33Leu)
		Glyma.06G210700	12	3	35	p.(Glu40Lys), p.(Glu27Lys), p.(Ala23Thr)
		Glyma.06G210800	38		8	
		Glyma.06G210900	11	1	36	p.(Gly463Arg)
		Glyma.06G211000			33	
		Glyma.06G211100	64			
		Glyma.06G211200	75		32	
		Glyma.06G211300	8		52	
		Glyma.06G211400	182	2	41	p.(Ala196Thr), p.(Ala200Thr)
		Glyma.06G211500	26	2	7	p.(Tyr124His), p.(Arg48Lys)
		Glyma.06G211600	36		12	
		Glyma.06G211700	11	7	18	p.(Ser140Ile), p.(Arg129His), p.(Arg58Lys), p.(Gly53Arg), p.(Arg50Gln), p.(Ala9Val), p.(Ser4Pro)
		Glyma.06G211800	9	2	10	p.(Tyr112Asn), earlystop p.(Gln222*)
		Glyma.06G211900		3	7	p.(Leu543Ser), p.(Ile307Val), p.(Asp221Asn),
		Glyma.06G212000	20			
**PC2**	***qPC2-2***	Glyma.06G203800	37	3		p.(Val131Gly), p.(Val193Gly), p.(Val211Gly)
		Glyma.06G203900	18		1	
		Glyma.06G204000	21	5	32	p.(His62Asn), p.(Asn107Tyr), p.(Leu159Phe), p.(Leu189Trp), p.(Ala565Thr)
		Glyma.06G204100	8	2	45	p.(Ala84Ser), p.(Glu9Asp)
		Glyma.06G204200	7	2	3	p.(Cys7Ser), p.(Val187Ile)
		Glyma.06G204300	4	1	5	p.(Thr229Ser)
		Glyma.06G204400	18	6	23	p.(Pro669Leu), p.(Pro673Leu), p.(Val519Ile), p.(Val523Ile), p.(Gly343Ala), p.(Gly347Ala)
		Glyma.06G204500	17		17	
		Glyma.06G204600		6	25	p.(Val588Ile), p.(Val866Ile), p.(Val935Ile), p.(Val966Ile), p.(Val1025Ile), p.(Val1035Ile)

Fig. S1. Seed coat colors of the parental lines Jinpung and IT109098 and a mapping population of recombinant inbred lines (RILs) derived from Jinpung x IT109098. (A) Left: Jinpung with a yellow seed coat color; right: IT109098 with a greenish-brown seed coat color. (B) RILs with varying seed coat colors. Scale bar: 10mm

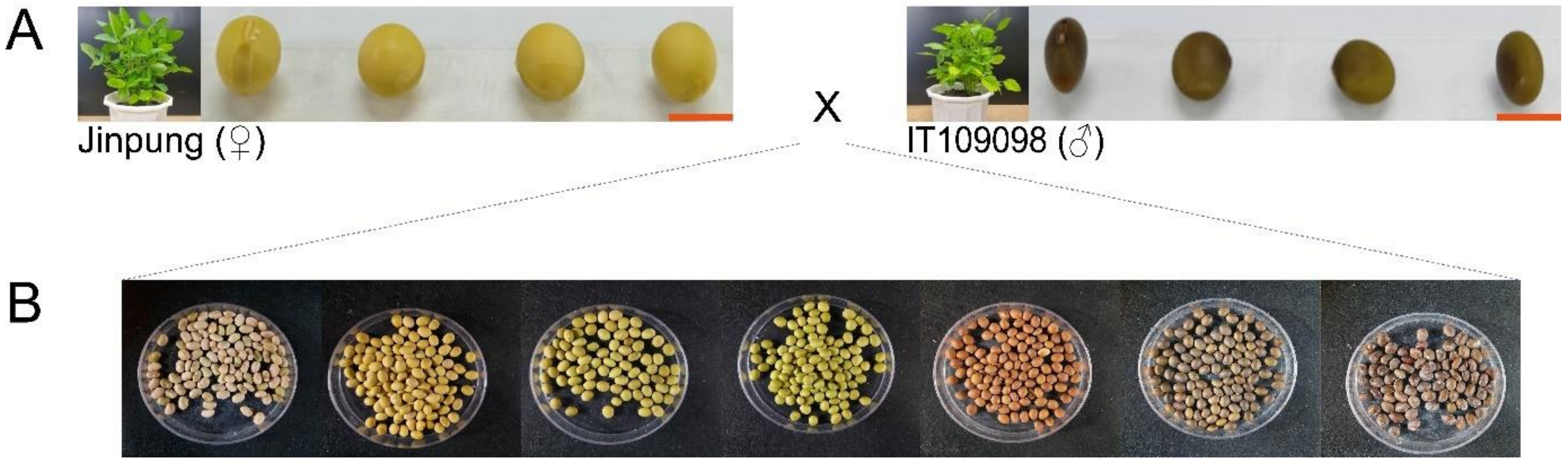

Fig. S2. Correlation among L*, a*, b* and epicatechin content (EC). ***: $P \leq 0.001$.

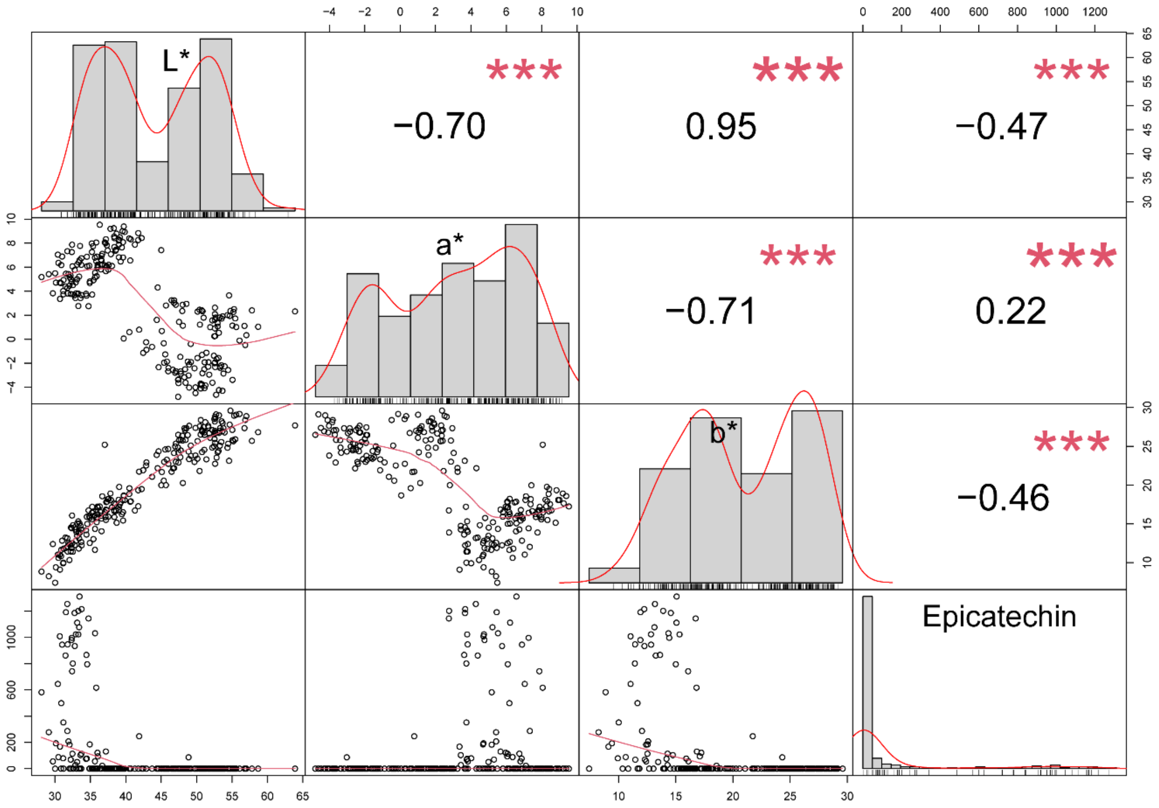

Fig. S3. Scatter plots of PC (PC1 and PC2) values against EC content

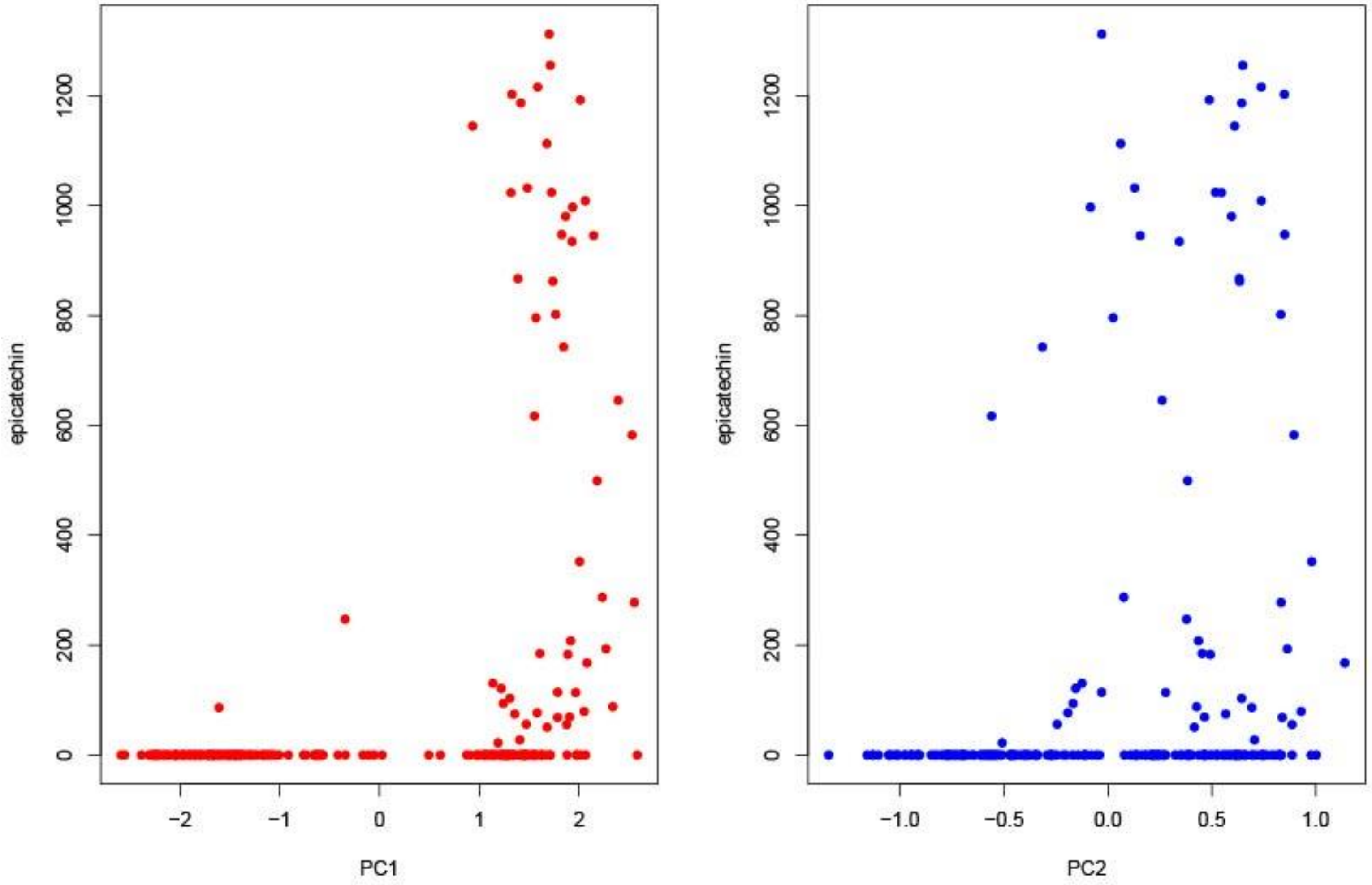

Fig. S4. Linear regression of PC1 and PC2 values against EC content of brown seeded RILs

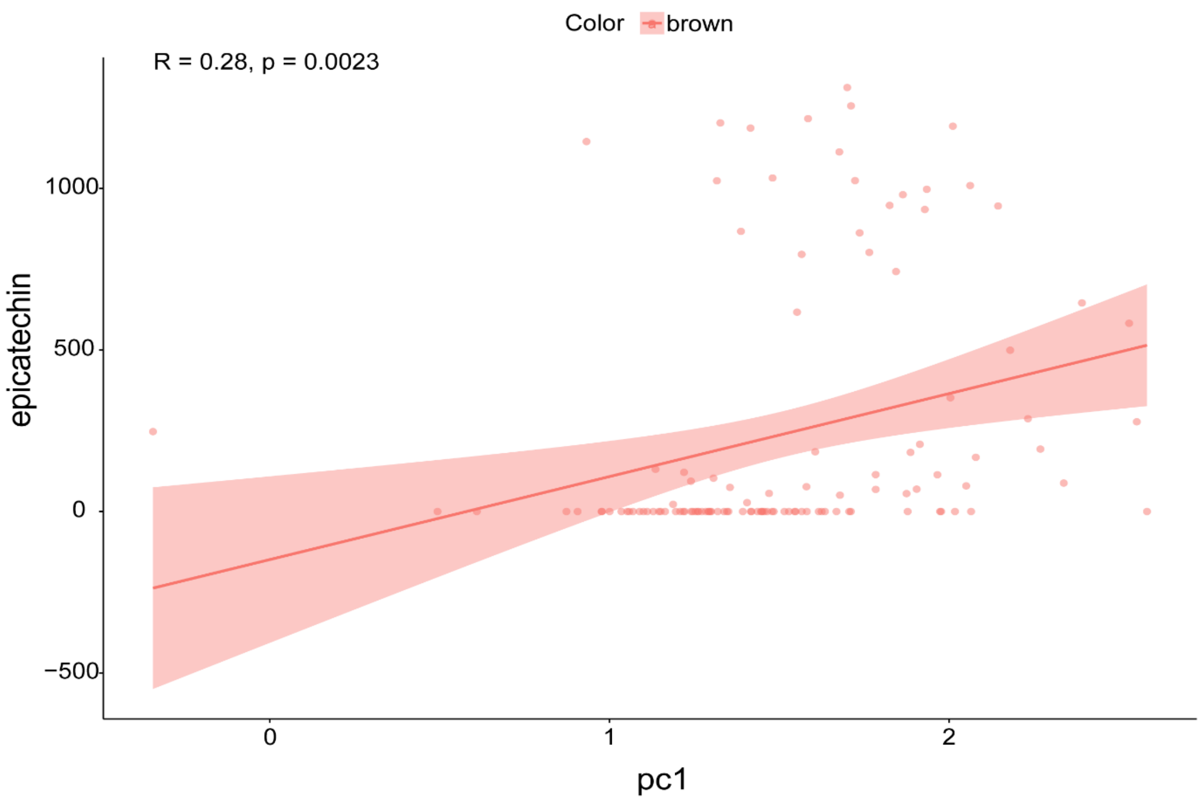

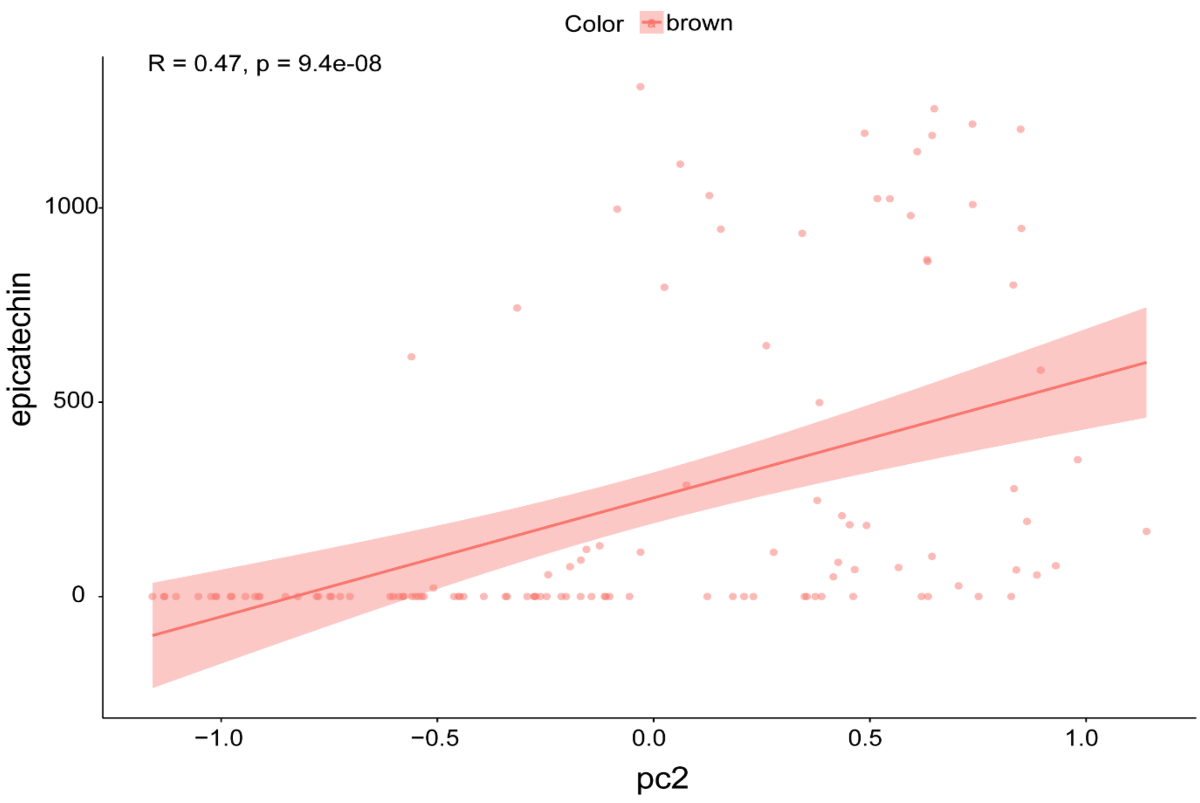

Fig. S5. Boxplots in ranges of PC1 and PC2 values against EC content. (A) 5 groups of PC1 ranges and their EC content. (B) 5 groups of PC2 ranges and their EC content. *: $P \leq 0.05$; ***: $P \leq 0.001$.

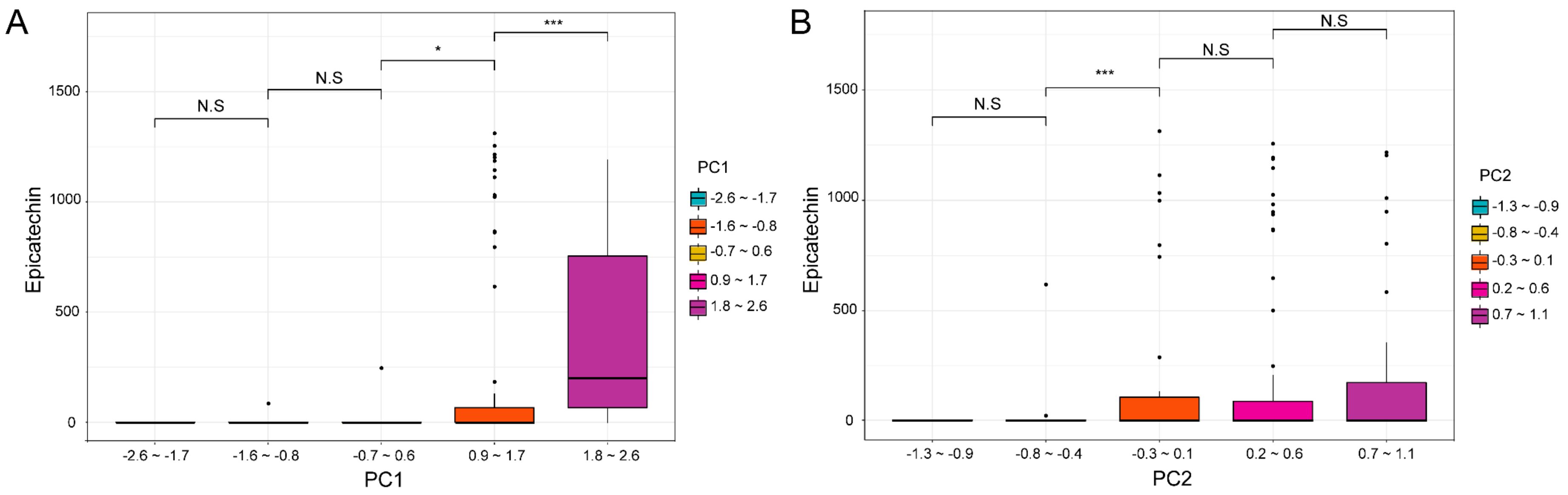

Fig. S6. Heatmap of gene expression within QTL *qL19-1*.

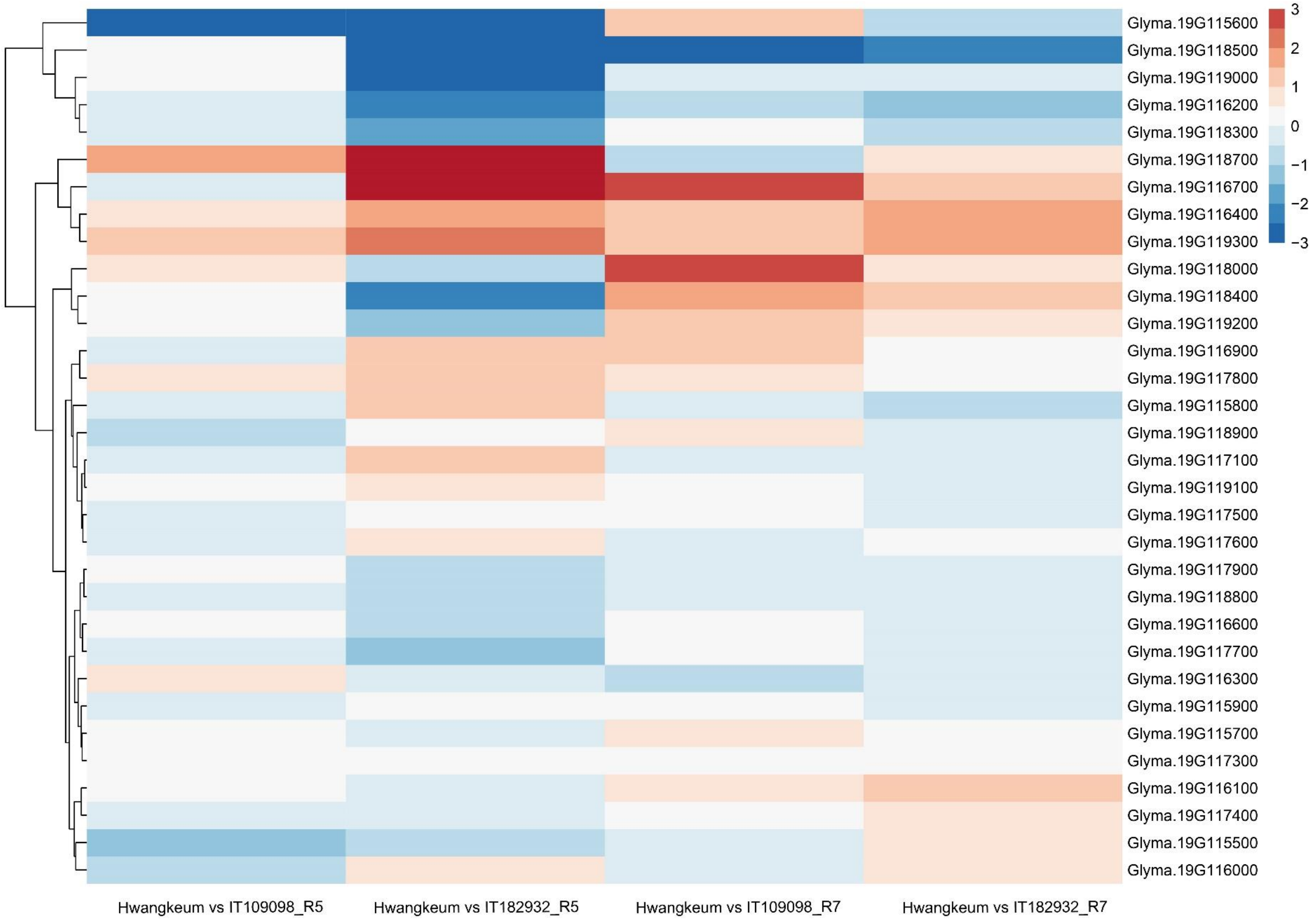

Fig. S7. Heatmap of gene expression within QTL *qEC01*.

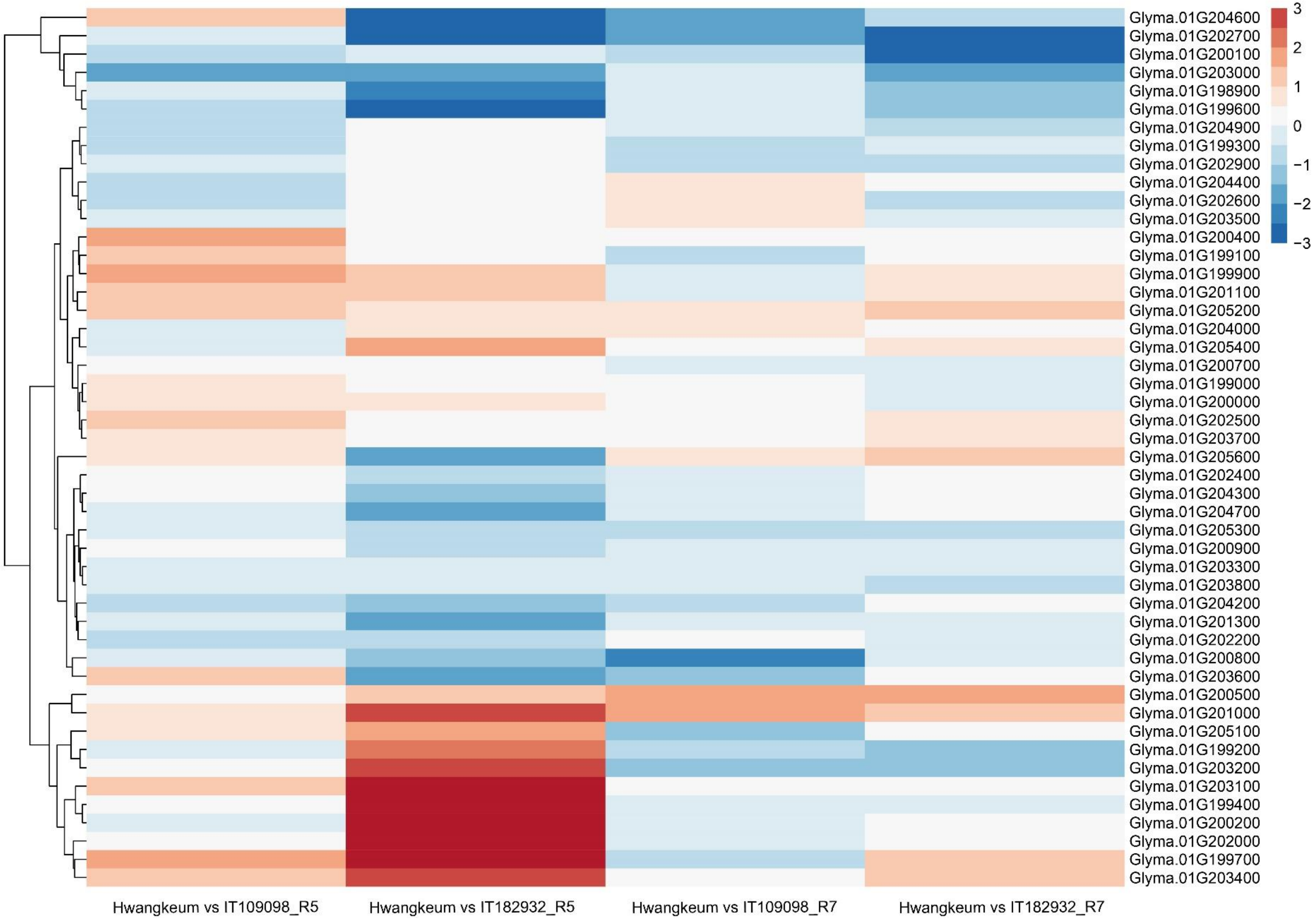

Fig. S8. Heatmap of gene expression within QTL *qB6-1*.

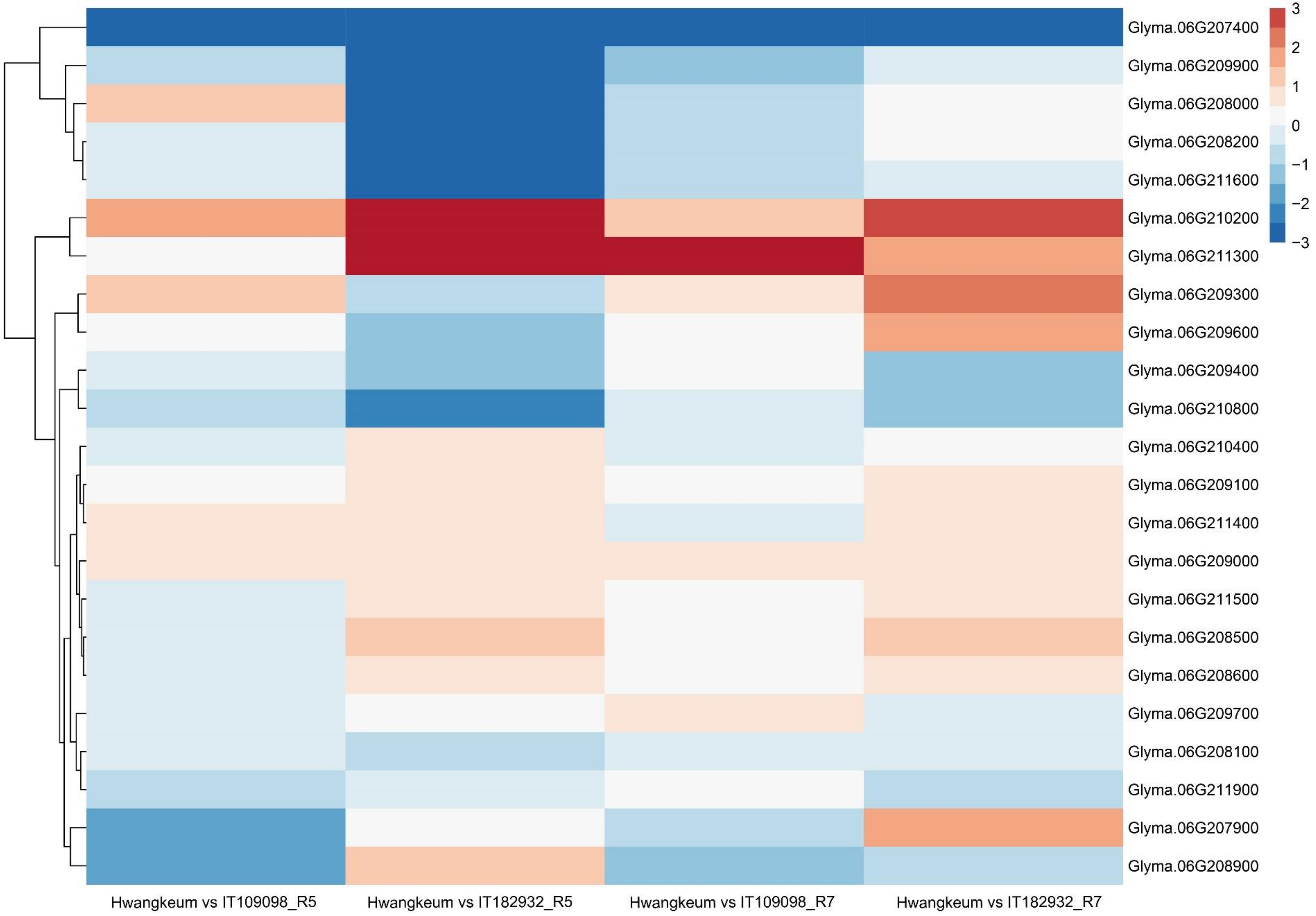

Fig. S9. Heatmap of gene expression within QTL *PC2-2*.

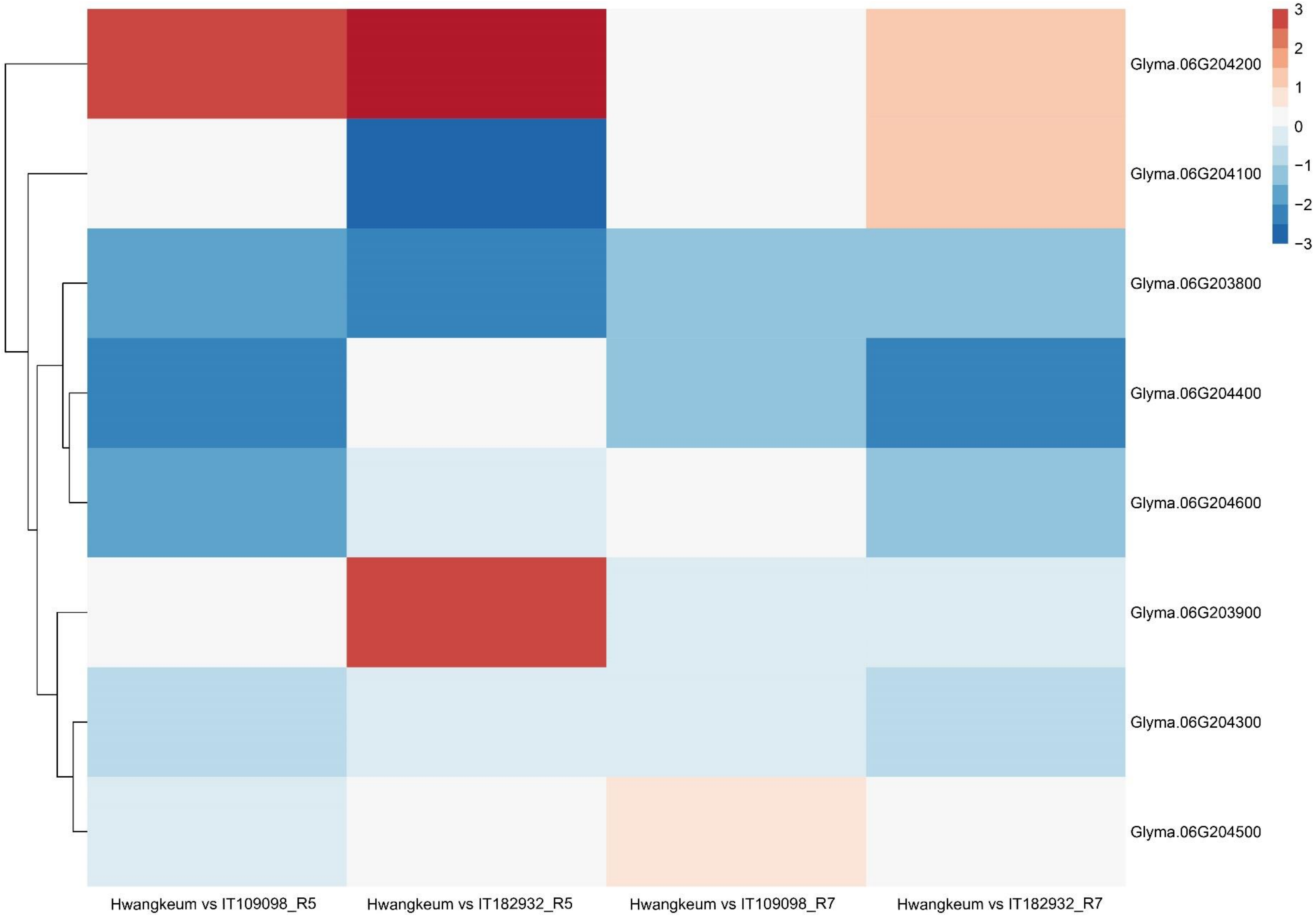

Supplement: Supplementary file 1 — Supplementary Material 1. [file 11032_2026_1655_MOESM1_ESM.pdf]
